# Extraction and mapping of downpour impact and their Cumulonimbus origin, 20 May 2020, Vâlcea (Romania) via Sentinnel-1 SAR dual polarization

**DOI:** 10.1038/s41598-022-22909-3

**Published:** 2022-11-14

**Authors:** Kamel Hachemi, Florina Grecu, Dana Maria Constantin (Oprea), Gabriela Ioana-Toroimac

**Affiliations:** 1 Laboratory MEDIATIONS, Sorbonne University and Paris-Est University, Maison de la Recherche, 28 Rue Serpente, 75006 Paris, France; 2 Faculty of Geography, University of Bucharest, 1 Nicolae Bălcescu Boulevard, 010041 Bucharest, Romania

**Keywords:** Climate sciences, Environmental sciences, Hydrology, Natural hazards

## Abstract

The aim of this work is to study the impact and characteristics of the meteorological phenomenon that occurred on May 20, 2020 in Vâlcea County, Romania. For this purpose, we used SAR radar images from the Sentinel-1 series at different dates, before, during and after the event. The methodology consists in exploiting and combining the two polarisations, VV and VH, of the recorded radar wave. The results obtained can be summarised as the extraction of areas completely covered by water and areas characterised by high roughness and very high humidity. The latter (roughness/humidity) can have two different origins. The first one corresponds to an area affected by downpours, giving a high roughness due to the interaction of water drops with the ground and also to the presence of hail, amplified by the wind factor. On the other hand, the second one coincides, quite simply, with the presence of a thundercloud, precisely a Cumulonimbus, which formed in that particular place as a result of the favourable geomorphological characteristics and meteorological conditions, giving a high humidity due to the high water content. We also determined the total impacted area of about 96.71 km^2^, whose 60.17 km^2^ of water covered area, which is 2.45% of the study area. The remaining 36.54 km^2^ (1.49%) represents the affected rough surface, located in the plain, or the humid surface corresponding to the area of the Cumulonimbus head covering the plain.

## Introduction

The random nature of extreme events makes it difficult to determine their causes and predict when they will occur. The impact on the population and the environment is estimated through quantitative data, assessments of risk elements (number of deaths, material damage, destruction in the living and natural environment, etc.). The cartographic representation of vulnerability to extreme events, i.e. the vulnerability map (and risk map) is a qualitative map^1^. The present study focuses on the method starting from the satellite images recorded on the dynamics of extreme weather phenomenon and the immediate impact, in short time, on the land surface (see “Study area”).

The objective of this work is to study the natural phenomenon that occurred on May 20, 2020 in a hilly region located in south central Romania, between the Southern Carpathians and the Romanian plain and to extract and map its impact and understand its origin. Administratively, the region is part of Wallachia. The analysis is made over a longer period and over a large area to understand the genesis and evolution of the regional phenomenon.

The region of Wallachia^2^ and especially the Oltenia (Lesser Wallachia bounded by the Olt river to the East), bounded by the Danube to the South and the Carpathians to the North, is characterized by a temperate continental climate with a Mediterranean influence from the South to the West)^3^. It is exposed to several climatic and meteorological risks, floods (2013), torrential rains, snowstorms, electrical storms (atmospheric disturbance)^4^.

Usually during an extreme weather event, optical images are not usable due to cloud cover. To remedy this problem, SAR (Synthetic Aperture Radar) images are used thanks to their operation all weather, day, night and regardless of meteorological conditions. The recorded radiometry gives information on the dielectric (soil moisture) and geometric (roughness) characteristics of the imaged surface.

Since the launch by ESA (European Space Agency) of the series of Earth observation satellites, Sentinel-1, Sentinel-1A (April 3, 2014) followed by Sentinel-1B (April 25, 2016), several works have been accentuated in several domains, especially in the mapping of temperate zones following the climatic and meteorological hazards observed, such as, the estimation of sea wind speed along the Iroise Coast in France^5^; the mapping and characterization of the hydrological dynamics of the Poitevin coastal marsh in France^6^; the mapping of floods in urban areas, 2018 floods of Ankara (Turkey)^7^; the study of the properties of the snowpack and the mapping of wet snow in the Northern French Alps^8^; the mapping of flash flood areas of eight upazilas in Sunamganj district, Bangladesh^9^; the mapping of the 2015 floods in the city of Chennai and the 2018 flood event of Kerala in India^10,11^; the mapping and monitoring of floods and their impact on the Ramganga River in the Ganges basin^12^; the mapping of extraordinary floods that occurred during the month of April 2018 in Ebro River (Spain)^13^; the monitoring and the optimization of flood mapping for regions of the lower Mekong basin in Vietnam^14,15^; the detection and mapping of floods due to incessant rains and rising water levels in the Rapti and Ghaghara rivers during the month of August 2017 in the state of Uttar Pradesh in India^16^; the mapping of floods in the San-Pédro river basin and of areas at risk of flooding in Grand-Bassam in Côte d'Ivoire^17,18^; the monitoring the impact of cyclonic events in Madagascar^19^; the mapping flooding caused by Tropical Cyclone Cempaka in the Gunung Sewu karst landscape^20^; the flood mapping and assessment of their impacts on the Sperchios river basin in Greece^21^; the mapping the flood Inundation in Xinxiang City, Henan Province China^22^.

To achieve our goal, we will use data from the Sentinel-1 satellite and this thanks to the penetrating capacity of the radar wave and its two acquisition modes in dual polarization, parallel polarization (VV) and cross polarization (VH). The use of different polarizations has been shown to be effective in studying soils, especially surface roughness and water content^23,24^.

The methodology adopted in this work is to exploit the two polarizations, VV and VH in order to discriminate, extract and map the different areas impacted by the phenomenon of 20 May 2020. To carry out this task, we will use three SAR radar images acquired at different dates, before, during and after this event occurred in the county of Vâlcea. The role of the two images acquired before and after, is to confirm and observe the changes caused in this period. They help us to determine the origins by ruling out other sources and keeping only that due to the phenomenon studied.

## Material and method

### Study area

#### Geographic location and population

**Figure 1:**
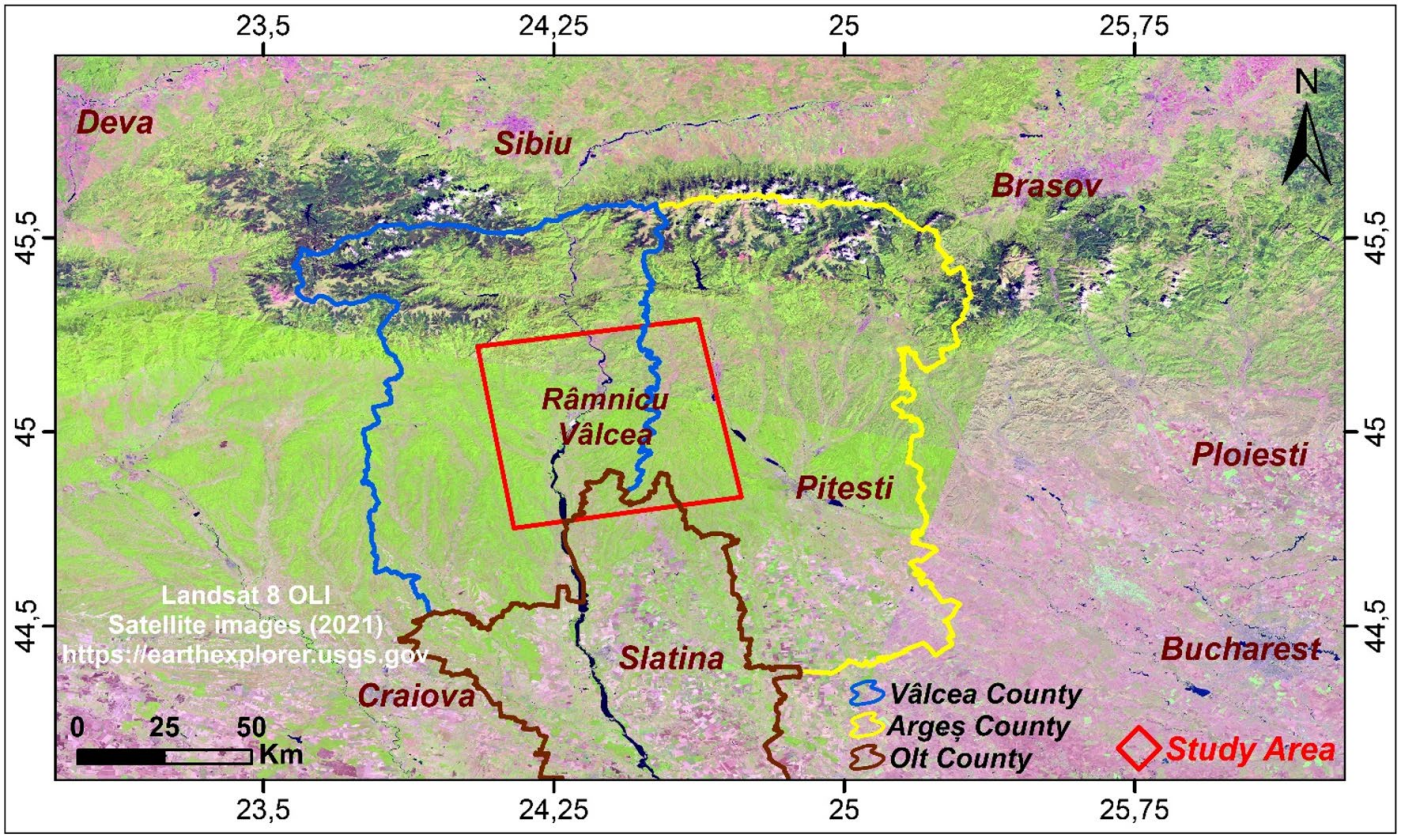
Geographical map generated from Landsat 8 OLI satellite images (2021) and produced using ArcGisPro 2.8.1, showing the study area (red frame) and the boundaries of the three counties, Vâlcea, Argeș and Olt. Source: https://earthexplorer.usgs.gov.

The study area is located northwest of Bucharest in the Wallachia region of Romania. It is bounded by longitudes: 24° 02′ 10.41″–24° 44′ 14.82″ East and latitudes: 44° 45′ 18.54″–45° 17′ 33.08″ North. It is shared between three counties, Olt, Argeș and Vâlcea which encompasses the largest part (Fig. 1).

The impact of extreme weather events on the population implies an analysis of dense regions. From this point of view, the study area is part of the densely populated subcarpathian region^3^. It occupies an area of 2458.16 km^2^ (Table 1).

**Table 1:** Coordinates of the center of the study area.

Center of the study area				Perimeter (km)	Area (km^2^)
UTM coordinates, zone 35 N		Geographical coordinates			
X (m)	Y (m)	Lon (°)	Lat (°)		
294,205.08	4,988,891.12	24.39	45.02	198.61	2458.16

The county of Vâlcea has a population of 371,714 inhabitants (2011) on an area of 5765 km^2^, a density of 64 inhab./km^2^. It has 9 towns and two municipalities, Râmnicu Vâlcea with 98,776 inhabitants (2011) and Drăgășani with 17,871 inhabitants (2011) (Table 2). Its demographic evolution is characterized by a rate of 25.77% from 1930 (295,560 inhabitants) to 2011. This county (Vâlcea) is crossed by the Olt River, one of the influential Danube. This Olt river separates two regions, in the West the Oltenia region, called Lesser Wallachia and in the East the Muntenia region, called Greater Wallachia.

**Table 2:** Towns MICI and their number of populations in Vâlcea County (2011).

Town	Băbeni	Călimănești	Horezu	Brezoi	Bălcești	Berbești	Băile Olanești	Ocnele Mari	Băile Govora
Population	8451	7622	6263	6022	4864	4836	4186	3309	2449

The Wallachia region represents the southern part of Romania. It is chartered by hills and plains that are spread out between the southern slopes of the Carpathians and the northern bank of the Danube.

#### Climatic and meteorological characteristics

This region is characterized by a continental climate, with very hot and stormy summers, and very cold and snowy winters with a slight warming observed in recent decades. The increase is distinguished especially in cities because of the decrease of the green spaces and the multiplication of the big buildings in glass and concrete.

Climatically, there are slight differences between the depression areas and the hills, but also between the submontane hills in the north and the piedmont hills in the south. It is located in the orographic shelter of the Carpathians and under the influence of some foehnal manifestations. The Olt corridor manifests itself as an area of ​​discontinuity between the eastern hills under the influence of the cold air of Arctic origin and the western hills, under the influence of some Mediterranean cyclones. This influence is felt in the hills of Oltenia to the Danube, in the appearance of local showers^25^. The relief of hills and depressions is felt in local weather events through thermal inversions and stormy phenomena—rain showers, hail storms^1,4,26^ with winter or summer effects. At the country level, the analyzed region is part of the area with over 40 days of thunderstorms; the maximum number of stormy days being 73 in Ramnicu Valcea, 81 in Polovragi, 77 in Targu Jiu, 79 in Drobeta Turnu Severin^1,26^. To the south, the number of days decreases, proof of the role of relief in the development of showers: 66 days in Targu Legresti, 61 days in Deagasani.

#### Geomorphological and geological data significant for the risk of meteorological manifestations

The analyzed region is part of the Getic Subcarpathians (sub-unit of the Valley Subcarpathians) and the Getic Plateau/Piedmont (Cotmeana Piedmont sub-unit), crossed from north to south by the Olt Corridor. The Subcarpathians and the Getic Piedmont make the transition between the high mountain unit in the north the Southern Carpathians or the Transylvanian Alps^2^ and the low plain unit in the South the Romanian Plain^27^.

The geomorphological peculiarities of the Subcarpathians, in line with those of the Carpathians, the presence of hills and depressions, morphometric and morphographic characteristics, position towards the development of high mountain peaks and towards the main baric centers are important factors in defining general climatic characteristics and short atmospheric disturbances duration. To the south, the Carpatho-Subcarpathian orogen makes the smooth transition to the Piedmont hills of the Getic Plateau following an initial evolution in writing^28^.

The main morphostructural units^29,30^ represent the synthesis of the evolution of the region (north–south) and the imprint of its current dynamics in the Subcarpathians.

The unity of the high hills (500–1000 m) and of the tectono-erosive submontane depressions overlaps the inscribe and inscribe formations (conglomerates, heavy, gravels, tuffs) generally monoclinic that appear in relief through the ridges. They are well developed east of Olt, where the steep hill fronts are oriented towards the mountain, and the inscribed surfaces tilt it slightly towards the Getic Piedmont. At contact there is a depressional relief on wide valleys. The unit is intensely affected by erosion and landslides. Morphometric parameters confirm the landscape of high hills with slopes of 25°–35°: the fragmentation density of the relief of 6–8 and 4–6 km/km^2^; fragmentation depth/relief energy of 250–300 and 200–250 m.

The unit of hills with average altitudes of 300–600 m with frequent slopes between 25° and 35° is defined by the Mi-Pliocene structures with diapir folds, anticline hills (Magura Slatioara-Govora-Ocnele Mari) and subcarpathian depressions (Ramnicu Valcea-Babeni, Horezu—Polovragi, Ocnele Mari; depression basins appear on the valleys, especially at confluences); Density fragmentation density 4–6 km/km^2^; fragmentation depth of 150–200 and 100–150 m.

The unit of low hills of 200–400 m presents a relief developed on monoclinic inscriptions deposits to the south, towards the Getic Piedmont, with gentle slopes, the fragmentation density of the relief of 2–4 km/km^2^; fragmentation depth of 0–50 and 50–100 m.

The relief with altitudes of 200–400 m can be followed along the valleys, with wide development on the Olt, Olanesti, Govora valleys. Most of the region has average values ​​of fragmentation of 4–6 km/km^2^ and relief energy of 150 and 200 m. Slopes with inclinations of over 25° and 35° have the highest spread due to the corrugated, monoclinic structure and diapirs.

In the valley and at the base of the slopes, the slopes are reduced, favoring the accumulation and stagnation of water and eroded materials on the slopes with high slope. Thus, the excess moisture appears in the areas, punctually.

The morphometric, morphographic and dynamic variations of the relief are well highlighted in analyzes on basins of rivers with springs in the Carpathians and discharge in the Romanian Plain, such as Oltetul^31^.

The Getic Piedmont, a unit of low hills, is presented as north–south oriented ridges, separated by valleys (500–600 m in the north, 200–240 m in the south). It consists of piedmont deposits (alternating gravel, sand, clay, marl) of Pleistocene age (Miocene and Pliocene have been identified by boreholes)^32^. The presence of permeable layers facilitates water infiltration and the appearance of springs and high-frequency torrents at the contact with the Roman Campia. In the Cotmeana piedmont, water infiltration leads to a lack of surface water on the interfluvial plateaus. The villages along the valleys benefit from groundwater from the alluvial basins. The impermeable layers and the deepening of the rivers favour landslides. Hypsometrically, it shows piedmont steps corresponding to alluvial–proluvial wedges^33^.

The Olt Valley forms a physical-geographical subunit that crosses the analyzed area from the north (Calimanesti) to the south (Dragasani, Slatina), in which the valley widens having a corridor aspect. In transversal profile, the following can be delimited: minor riverbed with the Olt River, minor riverbed (meadow), 6–7 levels of terraces, slopes. Along the Olt river dams and dykes were built (between 1970 and 1980) at Calimanesti, Daesti 209 ha, Ramnicu Valcea 319 ha, Raureni 174 ha, Govora 477 ha, Babeni 705 ha^29^; Ionesti 340 km^2^, Zavideni 156 km^2^, Dragasani 294 km^2^, Strejesti 527 km^2^, Aricesti 74 km^2^, Slatina 530 km^2^^3^. Artificial water accumulations diminish extreme phenomena in the riverbed, such as floods and floodwaters.

### Method

The methodology used in this work consists of extracting and mapping the different areas impacted by the meteorological phenomenon that occurred on May 20, 2020 and understanding its origin. The idea is to exploit by combining the two polarizations of the SAR radar wave, VV and VH to discriminate between areas completely covered with water and areas partially affected, characterized by high roughness and very high moisture (humidity).

Parallel polarization, VV is very effective for surface roughness studies because it is very sensitive to vertical elements. Cross polarization, VH is adequate in floodwater mapping^34^.

The concept is to amplify the weak signal by multiplying the backscattered coefficient of the two polarizations, σ0_VV_(db) × σ0_VH_ (db) to discriminate the water surfaces and to use the ratio σ0_VV_/σ0_VH_ to discriminate the rough and moist surfaces. We applied the same approach to the three SAR radar images acquired at different dates, before, during and after this event which occurred in this area of Vâlcea. The use of the two images acquired before and after is to confirm and observe the changes caused in this period. They help us to determine the origins by ruling out other sources and keeping only that due to the phenomenon studied.

### Data used

In this study, we used SAR (Synthetic Aperture Radar) radar images of the two satellites Sentinel 1A and Sentinel 1B at different dates, acquired respectively before, during and after the floods of 20/05/2020 (Table 3). These data are of the GRDH (Ground Range Detected High-resolution) production type and of the IW (Interferometric Wide) mode of Swath width 250 km with a resolution of 10 m, of C-band with wavelength 5.65 cm in double polarization, parallel polarization VV and cross polarization VH.

**Table 3:** Characteristics of the SAR radar data of the Sentinel-1A/B satellites used.

No.	Date	Hour (TIME) Start/stop	Satellite Sentinel	Orbite	Track	Pass	Centre Lat (°)/Lon (°)	Angle incidence (°)
1	14/05/2020	16:16:33/16:16:58	S1B	021580	29	Ascending	45.01/24.37	40.46°
2	20/05/2020	16:17:17/16:17:42	S1A	032651	29	Ascending	45.01/24.37	40.46°
2	01/06/2020	16:17:17/16:17:42	S1A	032826	29	Ascending	45.01/24.37	40.45°

Production type : GRD. Acquisition mode : IW. Mission : Sentinel-1. Polarisation VH et VV. Instrument abbreviation: SAR-C SAR.

In order to properly carry out this study, we are based on the weather data for each acquisition date (Tables 4, 5). We also used a DEM (Digital Elevation Model) of 12.5 m resolution, dated 14/01/2009, production ALOS-PALSAR (Advanced Land Observing Satellite—Phased Array type L- band Synthetic Aperture Radar), for the purpose of geometrical corrections of the data and to interpret the results.

**Table 4:** Weather conditions for each data acquisition used (Weather Station: Râmnicu Vâlcea).

No.	Date	Hour (hh:mm:ss)	Temperature (°C)	Average wind (km/h)	Max wind (km/h)	Dominant direction	Cloudiness (eighth)	Relative humidity (%)	Atmospheric pressure (hPa)
1	14/05/2020	16:16:33	28.3 (16:10) **28.1 (16:20)**	**8 (16:00)** 10 (17:00)	24 (16:10) **19 (16:20)**	SV	**5 (16:00)** 6 (17:00)	**42 (16:00)** 37 (17:00)	**983,9 (16:00)** 983,5 (17:00)
2	20/05/2020	16:17:17	21.2	**10 (16:00)** 7 (17:00)	16 (16:10) **12 (16:20)**	**NE (16:00)** NNE (17:00)	8	**68 (16:00)** 72 (17:00)	**981,7 (16:00)** 980,6 (17:00)
3	01/06/2020	16:17:17	21.2 (16:10) **20.8 (16:20)**	**11 (16:00)** 12 (17:00)	19 (16:10) **21 (16:20)**	NNE	5	**37 (16:00)** 38 (17:00)	**981,8 (16:00)** 981,9 (17:00)

Significant values are in bold.

**Table 5:** Daily quantities of precipitation at weather stations (WS) according National Meteorological Administration.

N°	Stations	Altitude h (m)	Geographical coordinates		Quantities of precipitation for each acquisition date (mm)		
			Lat	Lon	14/05/2020	20/05/2020	01/06/2020
1	Râmnicu Vâlcea	237.0	45°06′ 00″ N	24° 22′ 01″ E	0	48.6	0
2	Drăgășani	280.0	44°40′ 01″ N	24° 16′ 58″E	0	26	0.2
3	Târgu Logrești	262.0	45°55′ 01″ N	23° 43′ 58″ E	0	57.9	0
4	Târgu Jiu	204.0	45°01′ 58″ N	23° 16′ 01″ E	0.6	84.9	0
5	Polovragi	531.0	45°10′ 58″ N	23° 49′ 01″ E	1.2	55.6	0

### Treatments

**Figure 2:**
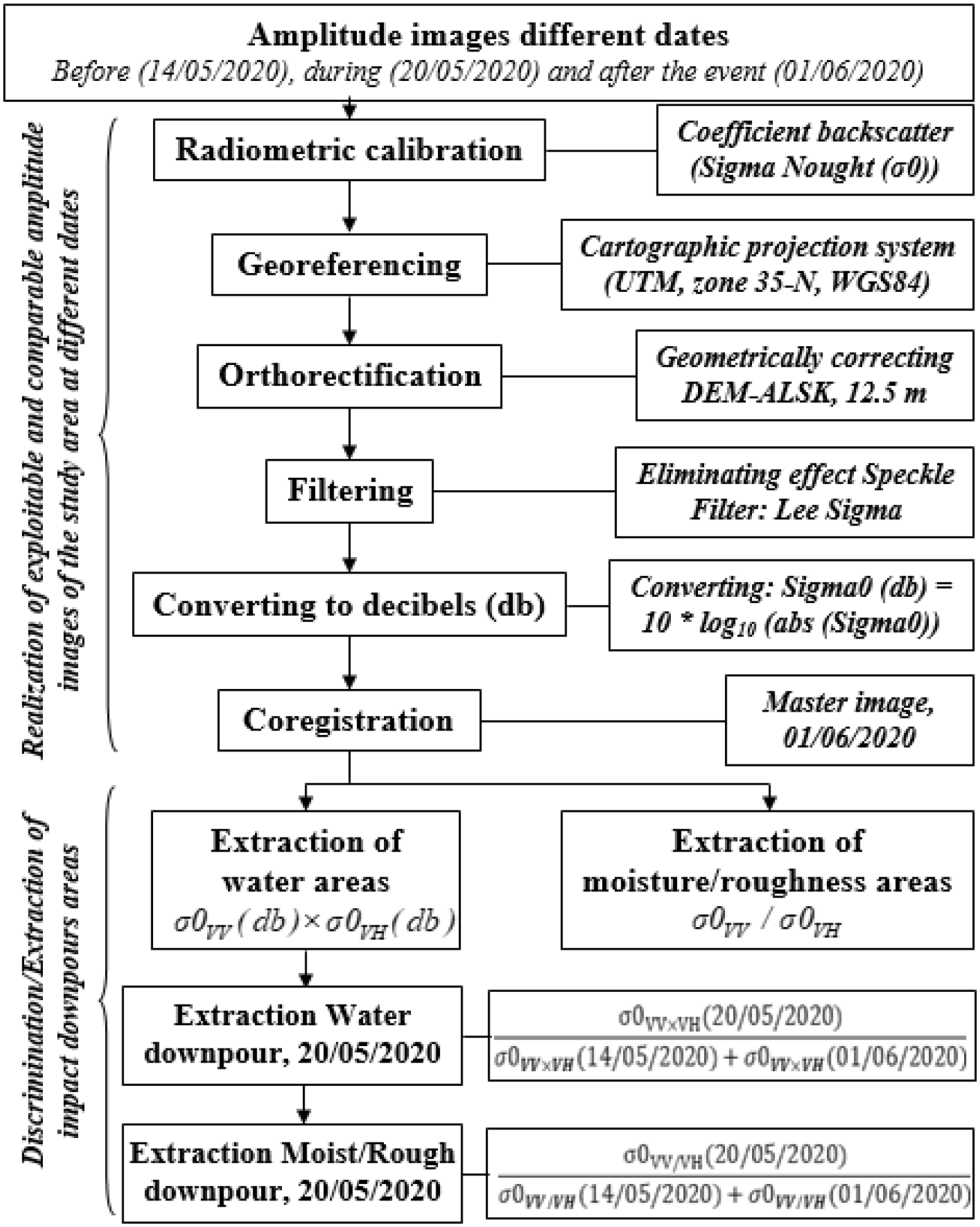
Flowchart showing the different steps of the performed treatments.

**Figure 3:**
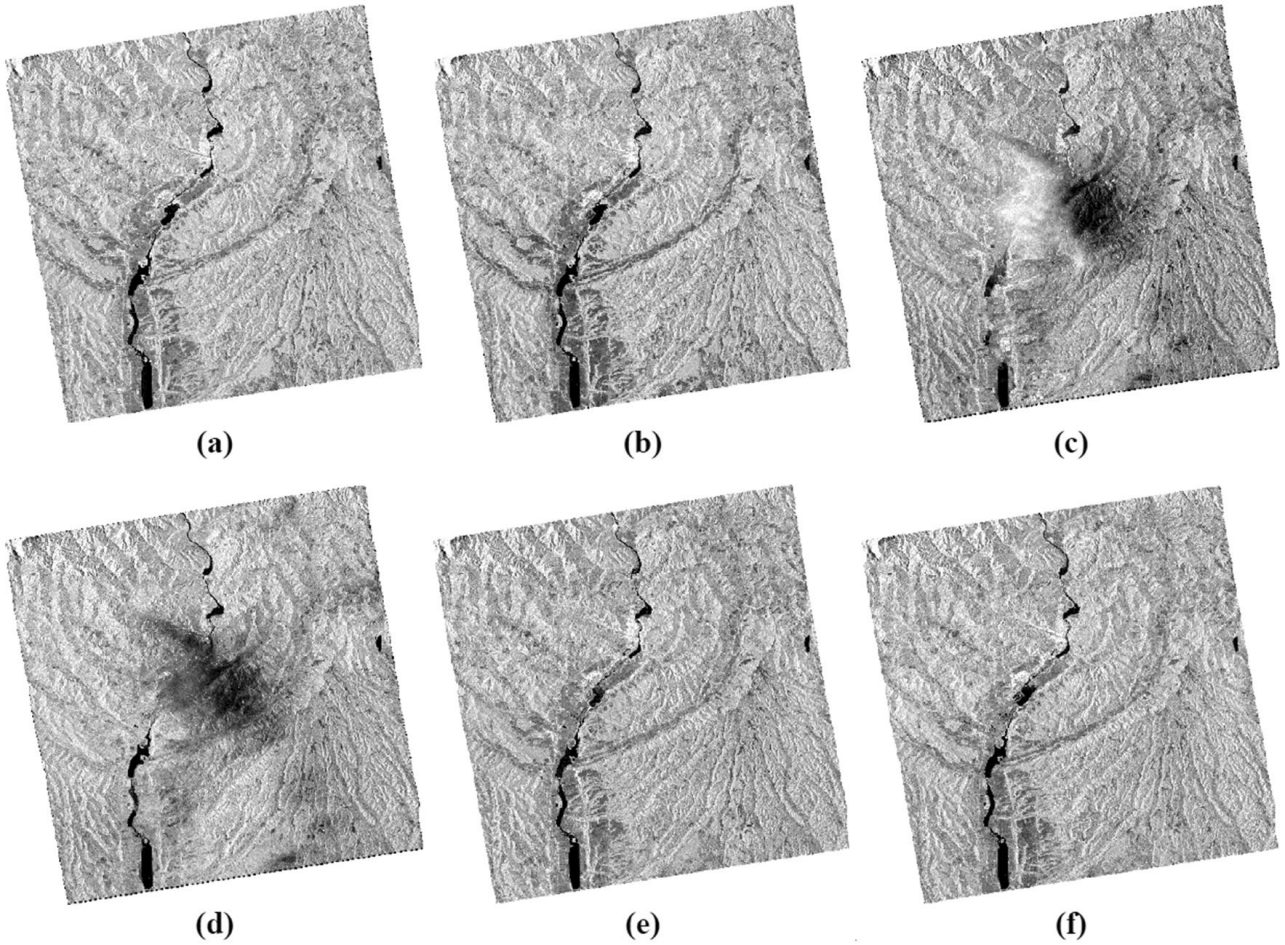
Amplitude images of the study area at different dates and different polarization, with the resolution of 10 m, calibrated, geo-referenced, ortho-rectified and filtered, (**a**) σ0_VV_ (db), 14/05/2020; (**b**) σ0_VH_ (db), 14/05/2020; (**c**) *σ*0_VV_ (db), 20/05/2020; (**d**) *σ*0_VH_ (db), 20/05/2020; (**e**) σ0_VV_ (db), 01/06/2020; (**f**) σ0_VH_ (db), 01/06/2020.

**Figure 4:**
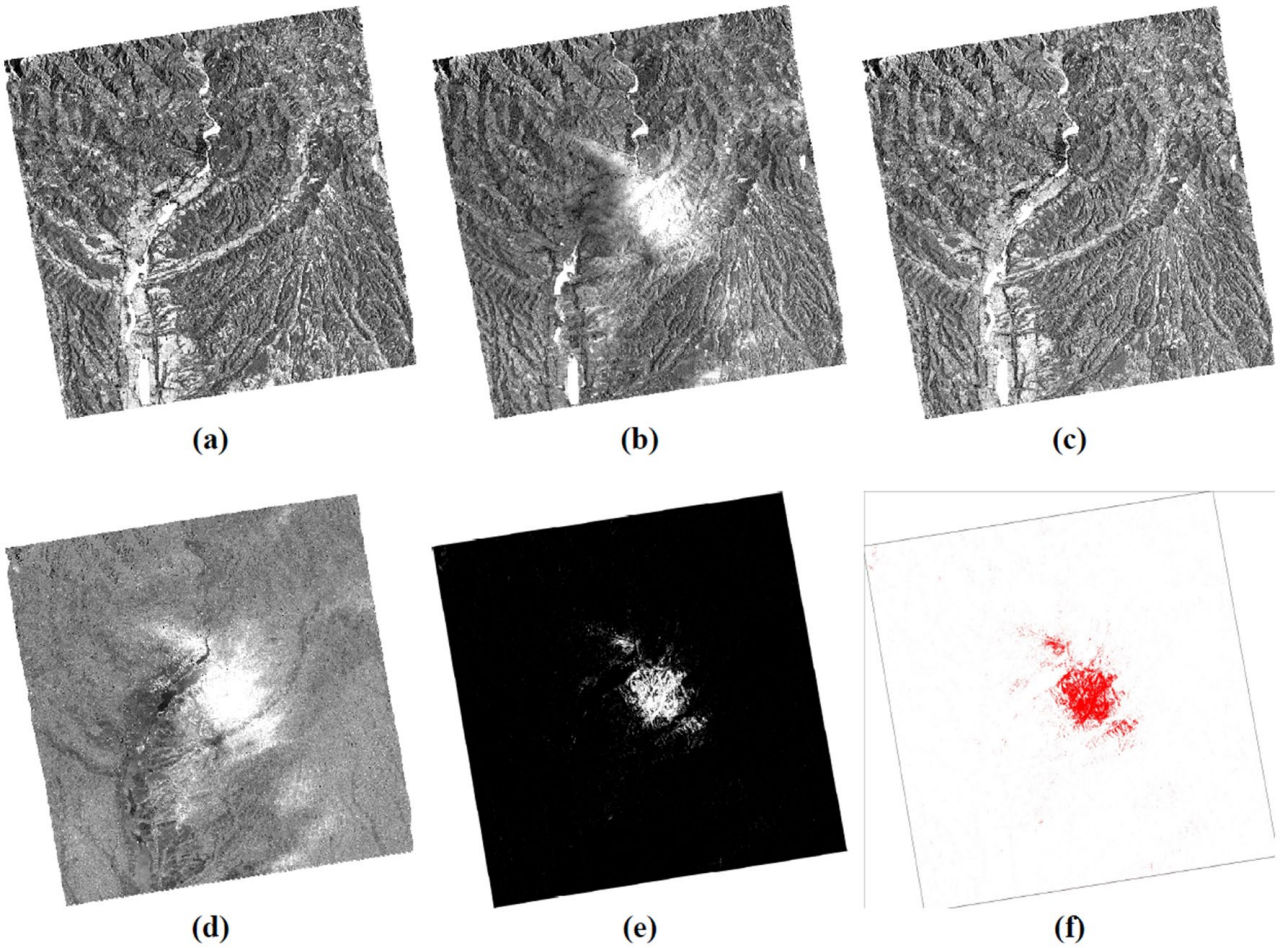
Steps for extraction of water surfaces from a VV and VH combination; (**a**) σ0_VVxVH_ (db), 14/05/2020; (**b**) σ0_VVxVH_ (db), 20/05/2020; (**c**) σ0_VVxVH_ (db), 01/06/2020; (**d**) extraction water downpour, 20/05/2020; (**e**) discrimination water downpour area, color white, 20/05/2020; (**f**) water downpour area, color red, 20/05/2020.

**Figure 5:**
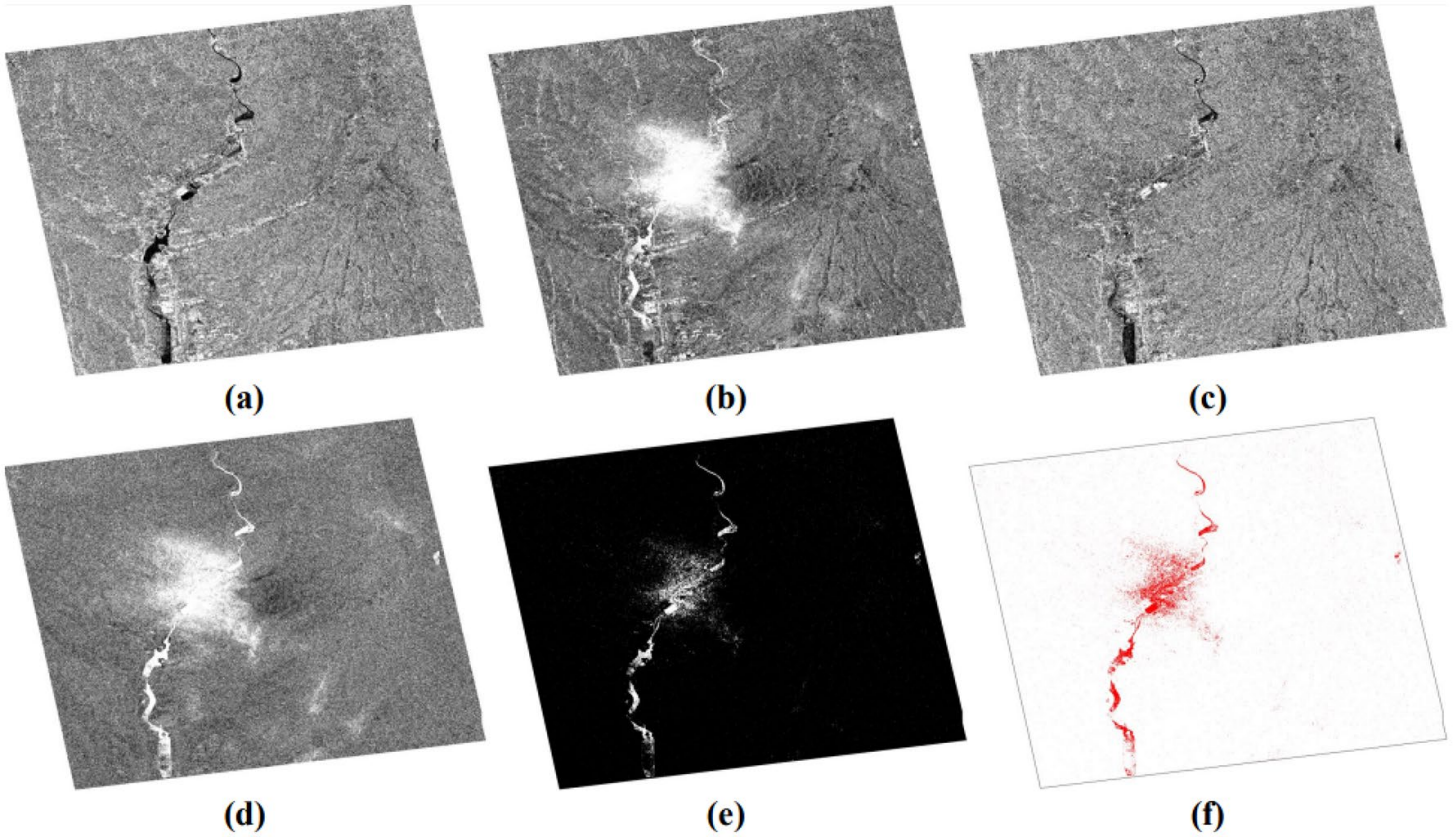
Extraction of high moisture/roughness surfaces from a combination of VV and VH; (**a**) σ0_VV/VH_, 14/05/2020; (**b**) σ0_VV/VH_, 20/05/2020; (**c**) σ0_VV/VH_, 01/06/2020; (**d**) extraction moisture/roughness downpour, 20/05/2020; (**e**) discrimination moisture/roughness downpour area, color white, 20/05/2020; (**f**) moisture/roughness downpour area, color red, 20/05/2020.

The treatments carried out can be summarized in two steps (Fig. 2): the first involves the production of images of usable and comparable amplitude of the study area at different dates in dual polarization, VV and VH (Fig. 3); the second focuses on the discrimination and extraction of impacted areas. The latter is distinguished by two purposes, the extraction of water-covered areas (Fig. 4) and the extraction of high moisture and strong roughness areas (Fig. 5).

### Ethics approval

All investigations relied on open access data.

## Results

**Figure 6:**
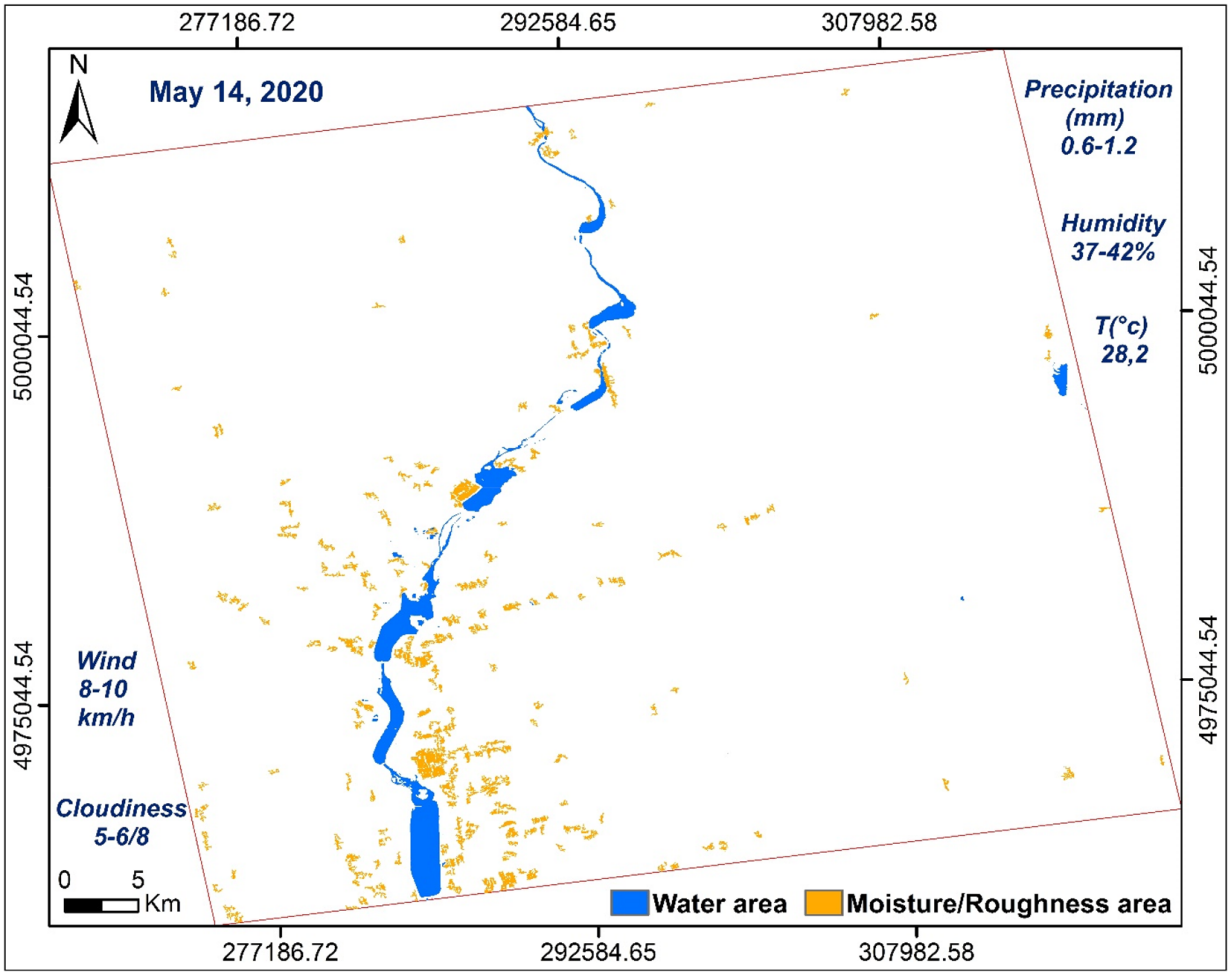
Map produced using ArcGisPro 2.8.1, showing areas of water and moisture/roughness, 14/05/2020.

**Figure 7:**
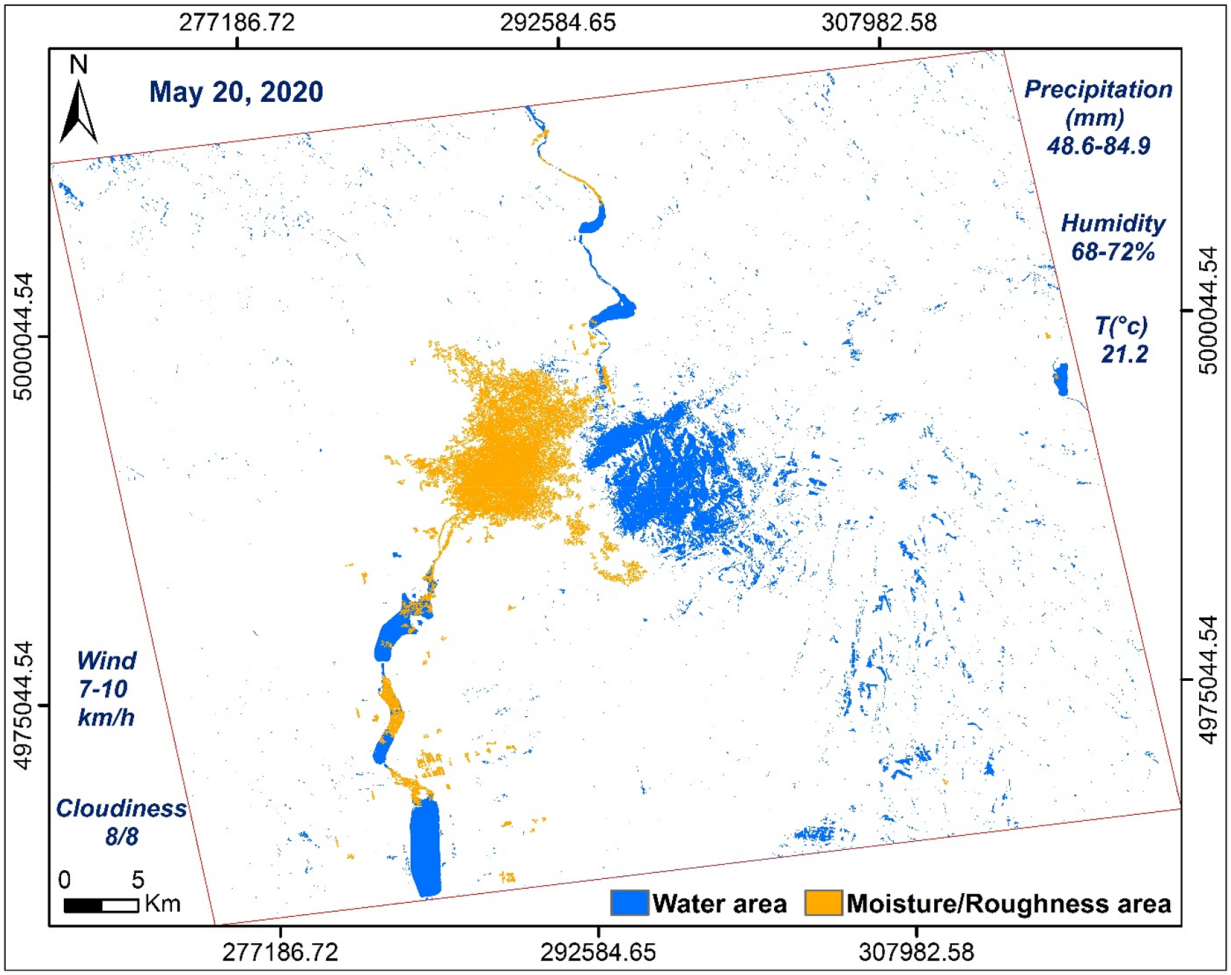
Map produced using ArcGisPro 2.8.1, showing areas of water and moisture/roughness, 20/05/2020.

**Figure 8:**
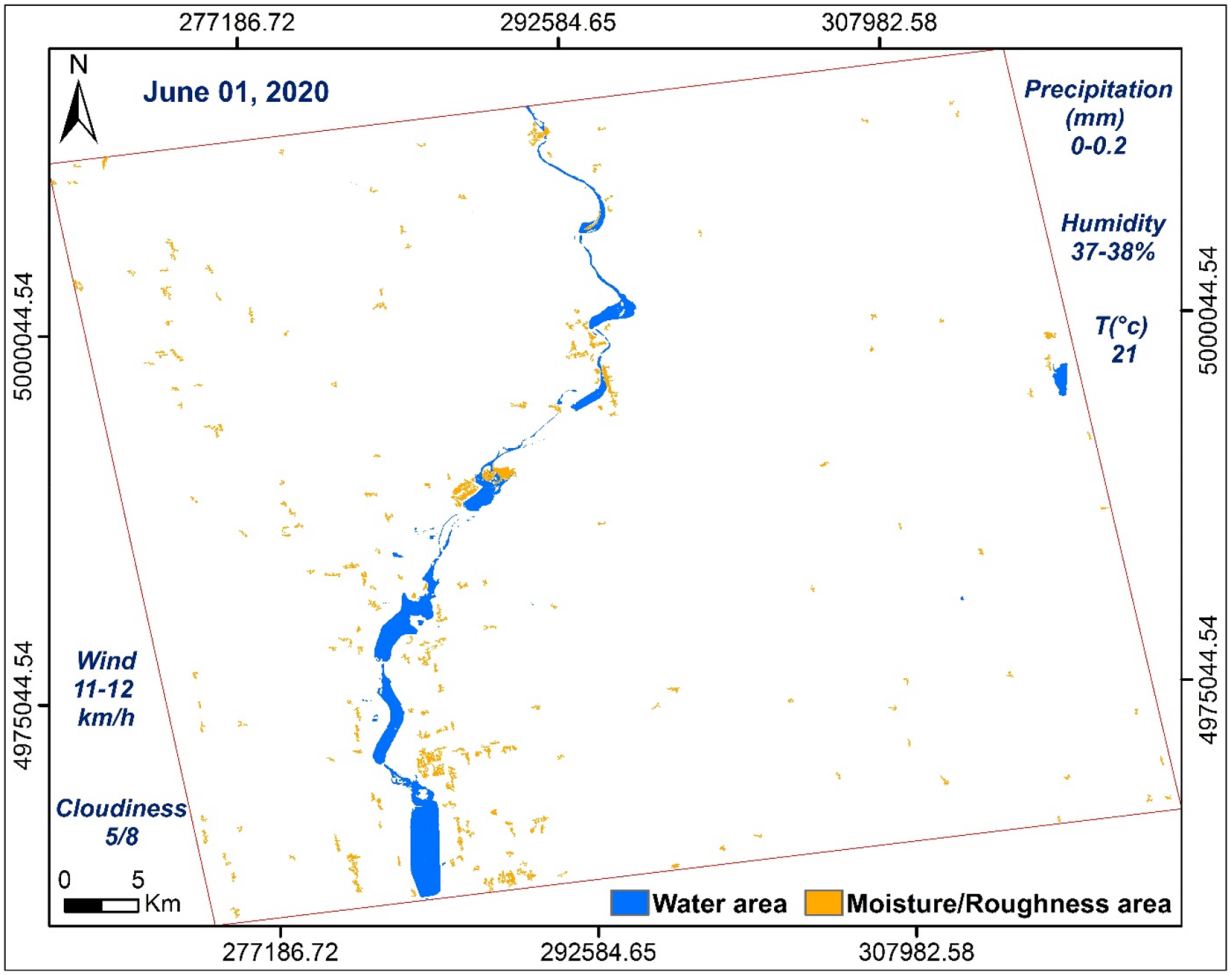
Map produced using ArcGisPro 2.8.1, showing areas of water and moisture/roughness, 01/06/2020.

Thanks to this study, we were able to produce maps showing the water surfaces at different dates (Figs. 6, 7, 8). We also estimated and calculated the areas of water zones (**S**_**W**_) and zones with very high humidity (moisture) and strong roughness (**S**_**M/R**_) of the three dates (Table 6).

**Table 6:** Results obtained from water surfaces and moisture/roughness at different dates.

	All surfaces at different dates: water surface (S_W_) and moisture/roughness surface (S_M/R_)					
Date	14/05/2020		20/05/2020		01/06/2020	
Area	S_W_	S_M/R_	S_W_	S_M/R_	S_W_	S_M/R_
(Km^2^)	32.95	30.93	91.88	61.06	30.48	18.11
(%)	1.34	1.26	3.74	2.48	1.24	0.74

The roughness is a function and depends on the two parameters, wavelength and angle of incidence. In our case and according to the Rayleigh criterion, a surface is considered rough if its average surface unit is greater than 9.12 mm for an angle of incidence of 40.46°, i.e. between 7.38 mm for 20° and 9.99 mm for 46°^35^. On the other hand, moisture represents the water content of an imaged surface. It is defined by its dielectric constant, the higher it is, the less the wave penetrates into the soil and the stronger the backscattered intensity. It acts on the transmission and absorption of the signal. Thus, the dielectric constant determines the depth of penetration of the radar wave. An increase in moisture content increases the dielectric constant and enhances the gloss of the soil. Dry ground, on the contrary, will have a weak signal^35^.

**Figure 9:**
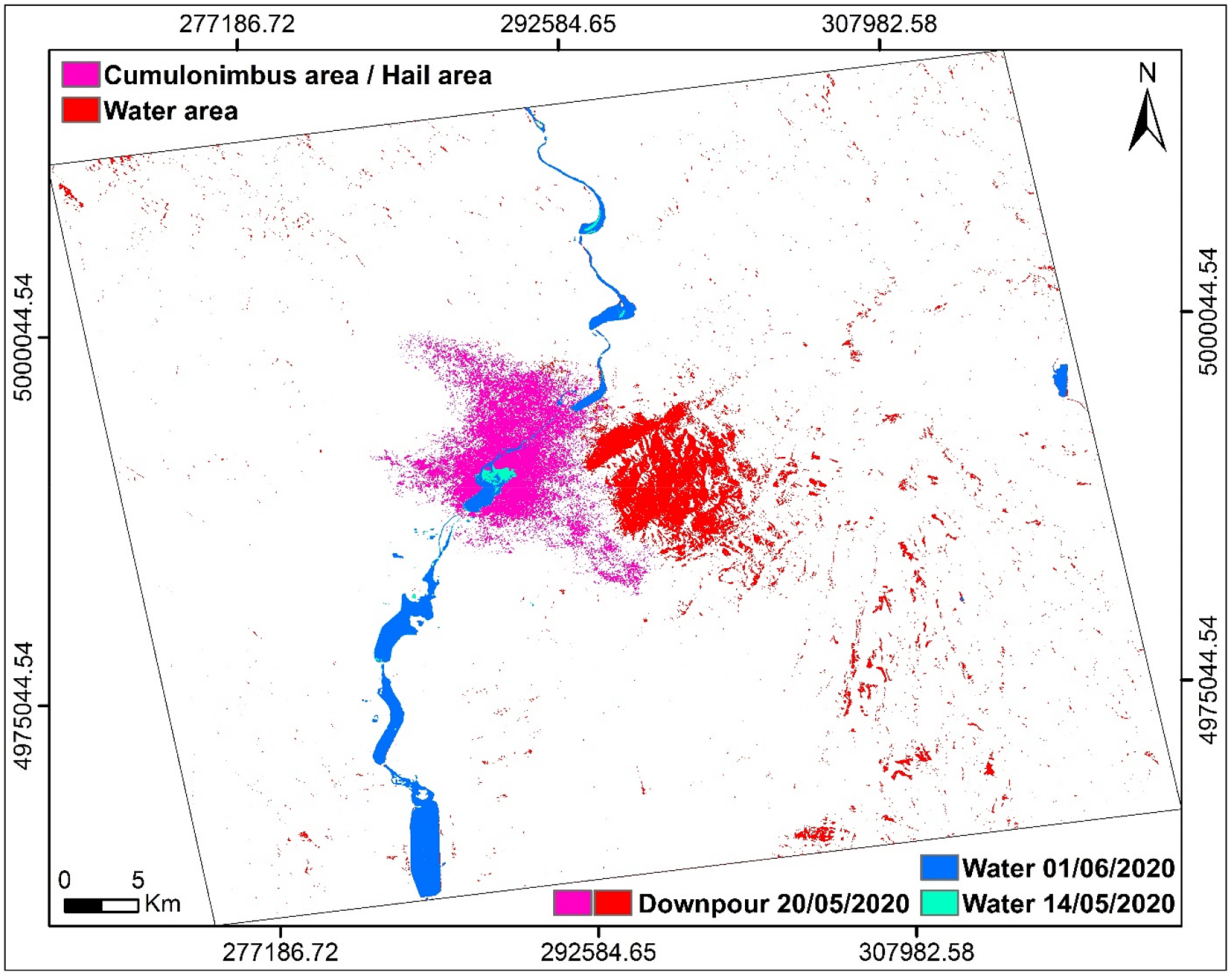
Downpour Impact map for 20/05/2020, produced using ArcGisPro 2.8.1.

We also produced a global map summarizing and showing the areas affected by the downpours of May 20, 2020 (Fig. 9). Subsequently, we calculated the surface areas of the areas affected by the downpours. The result corresponds to the evaluation of surfaces completely covered with water (**D**_**W**_) and of moist/rough surfaces affected by falling water drops and by hail, accentuated by the wind factor (**D**_**M/R**_) (Table 7).

**Table 7:** Results obtained from water surfaces and moisture/roughness surfaces at downpour of the 20/05/2020.

	Impact downpour surfaces: water (D_W_) and moisture/roughness (D_M/R_)	
Date	20/05/2020	
Area	D_W_	D_M/R_
(km^2^)	60.17	36.54
(%)	2.45	1.49

The estimate of this D_M/R_ area was calculated using the following formula (1). The humidity (moisture) is a variable in time, depending on the humidity rate and the roughness is constant, reflecting the geometric characteristics of the imaged surface, these two parameters must be eliminated to leave there only the parameters that are due to the events which are produced on 20 May 2020.

1 $${\text{D}}_{{\text{M/R}}} {\text{ = S}}_{{\text{M/R(e)}}} { - }\sum\limits_{{\text{i}}}^{{\text{f}}} {{\text{S}}_{{\text{M/R(i)}}} }$$

where ***e***: date 20/05/2020, ***i***: date 14/05/2020 and ***f***: 01/06/2020.

## Discussion

**Figure 10:**
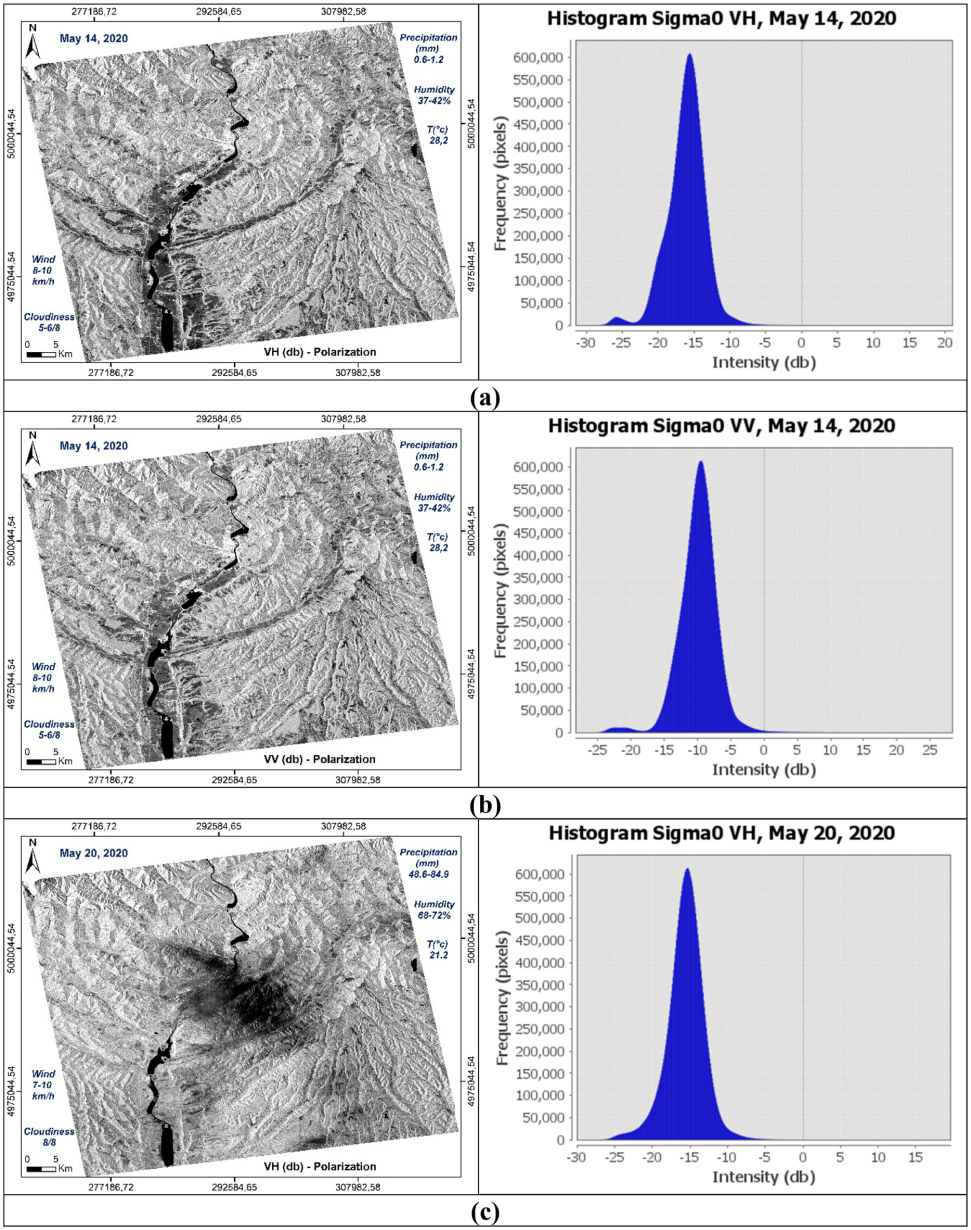

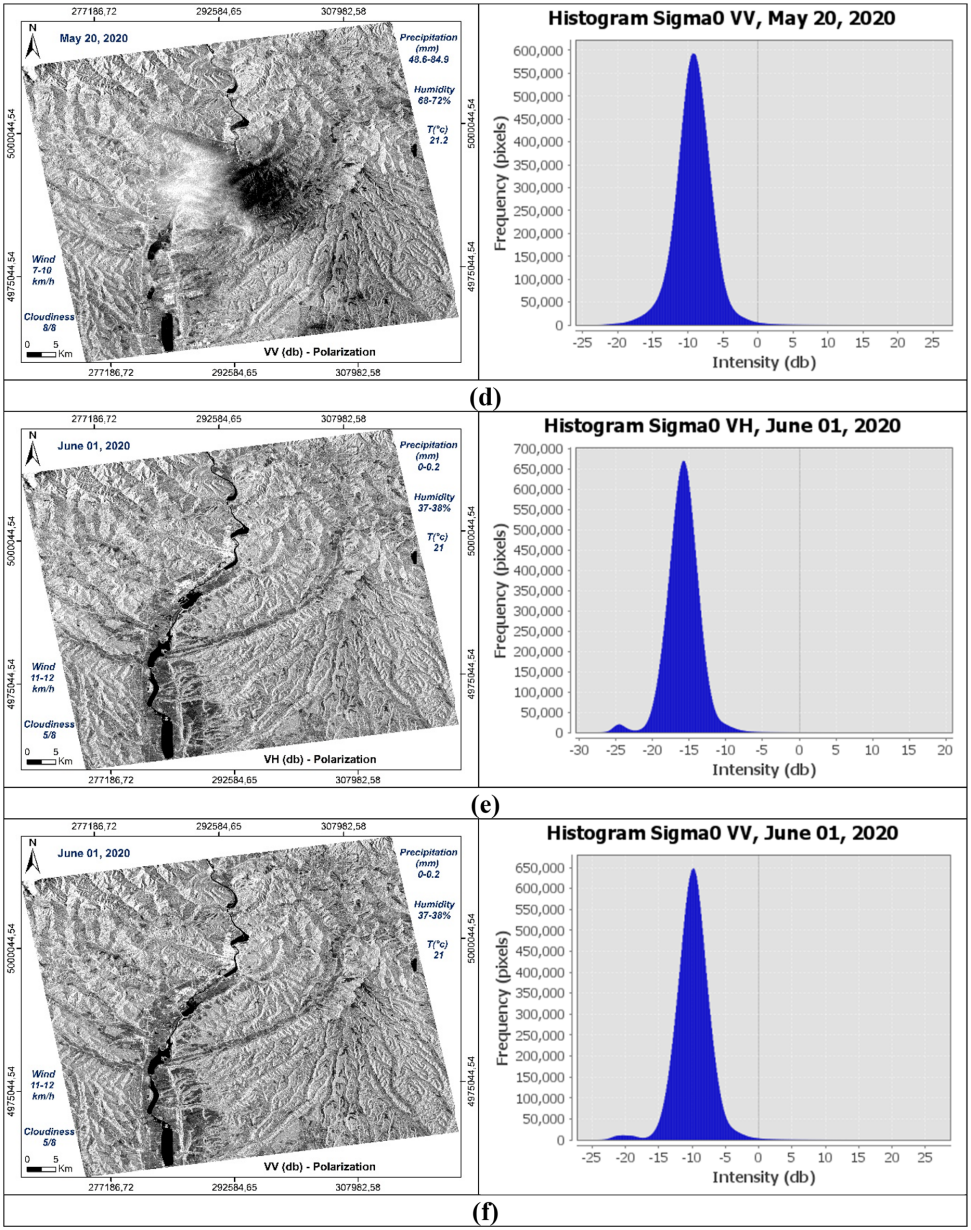
Histograms of the two polarisations VV and VH of the three SAR images used, dated before (14/05/2020), during (20/05/2020) and after (01/06/2020) the event.

The histograms of the recorded signal of the two polarizations VV and VH of the three images used, show a resemblance between the two dates before May 14, 2020 (Fig. 10a) and after June 01, 2020 (Fig. 10c) compared to the image acquired during the downpour of May 20, 2020 (Fig. 10b). Surfaces covered with water are well distinguished and have backscatter coefficient values ​​between -27 and -23 db on the cross band (**σ0**_**VH**_) (Fig. 10a,e), and in the parallel band (σ0_VV_) they are between -24 and -17 db (Fig. 10b,f). In these two cases, the restricted number of water pixels is well focused in the Olt River and other small places like Zigoneni Lake in Argeș (Fig. 10a,b,e,f). On the other hand, in the image acquired during the event, their values ​​are distributed because the water pixels are more numerous, thus covering more places following the downpours (Fig. 10c,d).

**Figure 11:**
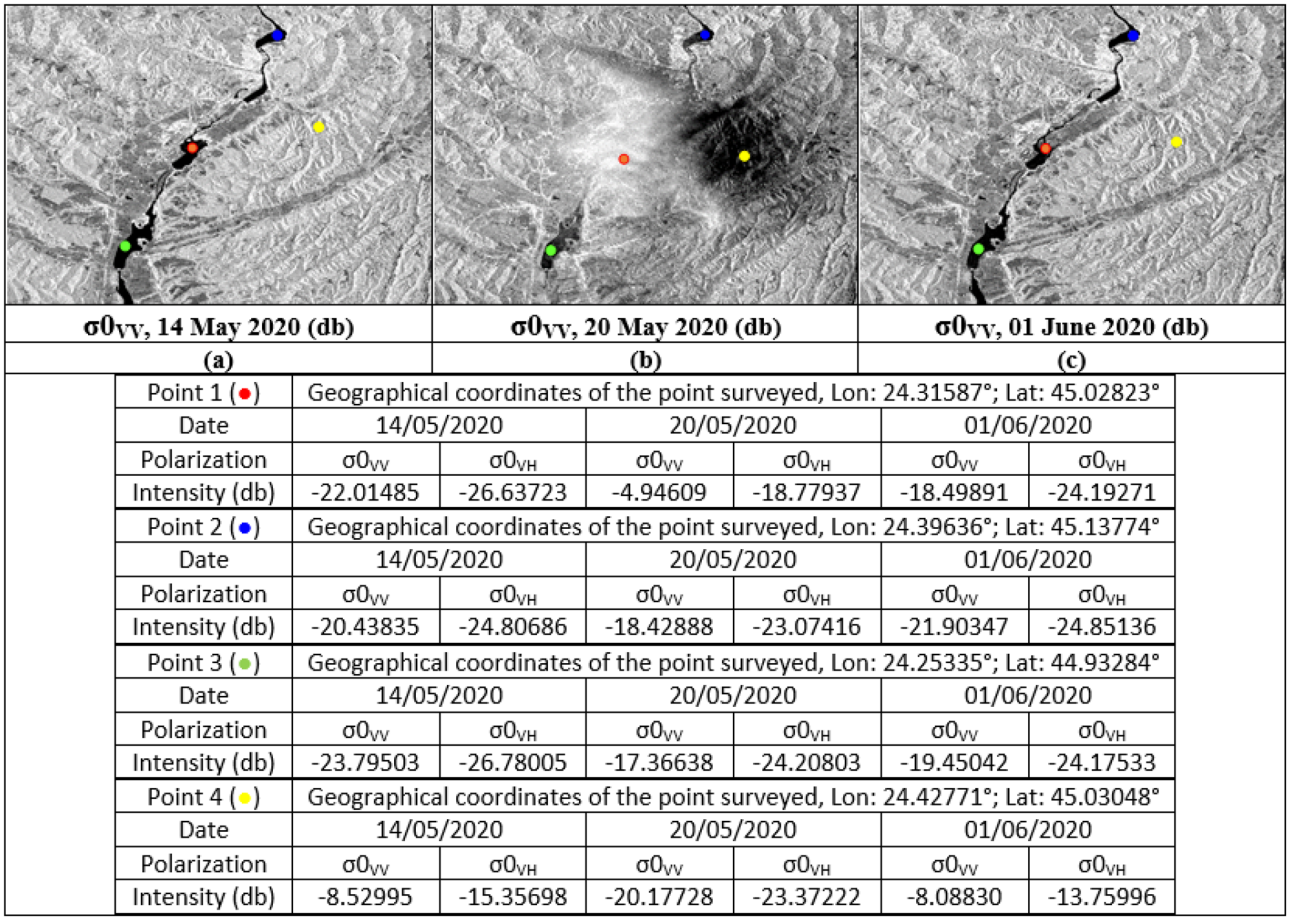
Images showing the points of the location at the level of the Olt River such as between the two banks Stupărie and Ruda for Point 1, (**a**) image before, dated May 14, 2020, VV polarization; (**b**) image during, dated May 20, 2020, VV polarization; (**c**) image after, dated June 01, 2020, VV polarization.

Concerning soil humidity and roughness, the two histograms before and after the event are almost identical. On the other hand, the image of May 20, 2020, the values ​​of the backscatter coefficient are between -10 and 0 db in the parallel band VV against -24 and -14 in the same locations for the two other images before and after in the same VV band which is sensitive to soil moisture and roughness (Fig. 10b,d,f). Especially in the locations where the water pixels are located like in the centre of the three images, i.e., at the level of the river of Olt between the two banks Stupărie and Ruda (Point 1, red colour), the values ​​of **σ0**_**VV**_ observed are -4.94609 db (20/05/2020) against -22.01485 db (14/05/2020) and -18.49891 db (01/06/2020) (Fig. 11). On the other hand, in the locations not impacted by the downpour, the values ​​do not represent the same differences as shown by Points 2 in the North (Blue colour) and Point 3 in the South (Green colour) in the table below (Fig. 11). Regarding the black area in the centre of the 20 May 2020 image is represented by pixels of low radiometry as shown in Point 4 (Yellow colour) compared to the two images of 14 May 2020 and 01 June 2020 which record high radiometry.

The extraction of water from the downpour of May 20, 2020, was carried out by amplifying the backscattered signal by the multiplication of its two polarizations VV and VH from the formula (2) of Water Extraction Downpour (**WE**_**D**_) for each date used (Fig. 4a–c) and using the formula (3) using the Water Change Detection Index in the case of Downpour (**WCDI**_**D**_) (Fig. 4d–f).

2 $${WE}_{D}= {\sigma 0}_{VV }\times {\upsigma 0}_{VH}$$

3 $${{\varvec{W}}{\varvec{C}}{\varvec{D}}{\varvec{I}}}_{{\varvec{D}}}=\frac{{\upsigma 0}_{\mathrm{VV}\times \mathrm{VH}}(20/05/2020)}{{\sigma 0}_{{\varvec{V}}{\varvec{V}}\times {\varvec{V}}{\varvec{H}}}(14/05/2020)+{\sigma 0}_{{\varvec{V}}{\varvec{V}}\times {\varvec{V}}{\varvec{H}}}(01/06/2020)}$$

For the extraction of the moisture and the roughness of the soil due to the downpour of May 20, 2020, the same procedure was followed but this time using the ratio of the backscattered signal of its two polarizations VV and VH for each date used (Fig. 5a–c) from formula (4) of Moisture/Roughness Extraction Downpour (**MRE**_**D**_) and also using formula (5) adapting the Moisture/Roughness Change Detection Index in case Downpour (**MRCDI**_**D**_) (Fig. 5d–f).

4 $${MRE}_{D}= {\sigma 0}_{VV }/{\upsigma 0}_{VH}$$

5 $${{\varvec{M}}{\varvec{R}}{\varvec{C}}{\varvec{D}}{\varvec{I}}}_{{\varvec{D}}}=\frac{{\upsigma 0}_{\mathrm{VV}/\mathrm{VH}}(20/05/2020)}{{\sigma 0}_{{\varvec{V}}{\varvec{V}}/{\varvec{V}}{\varvec{H}}}(14/05/2020)+{\sigma 0}_{{\varvec{V}}{\varvec{V}}/{\varvec{V}}{\varvec{H}}}(01/06/2020)}$$

**Figure 12:**
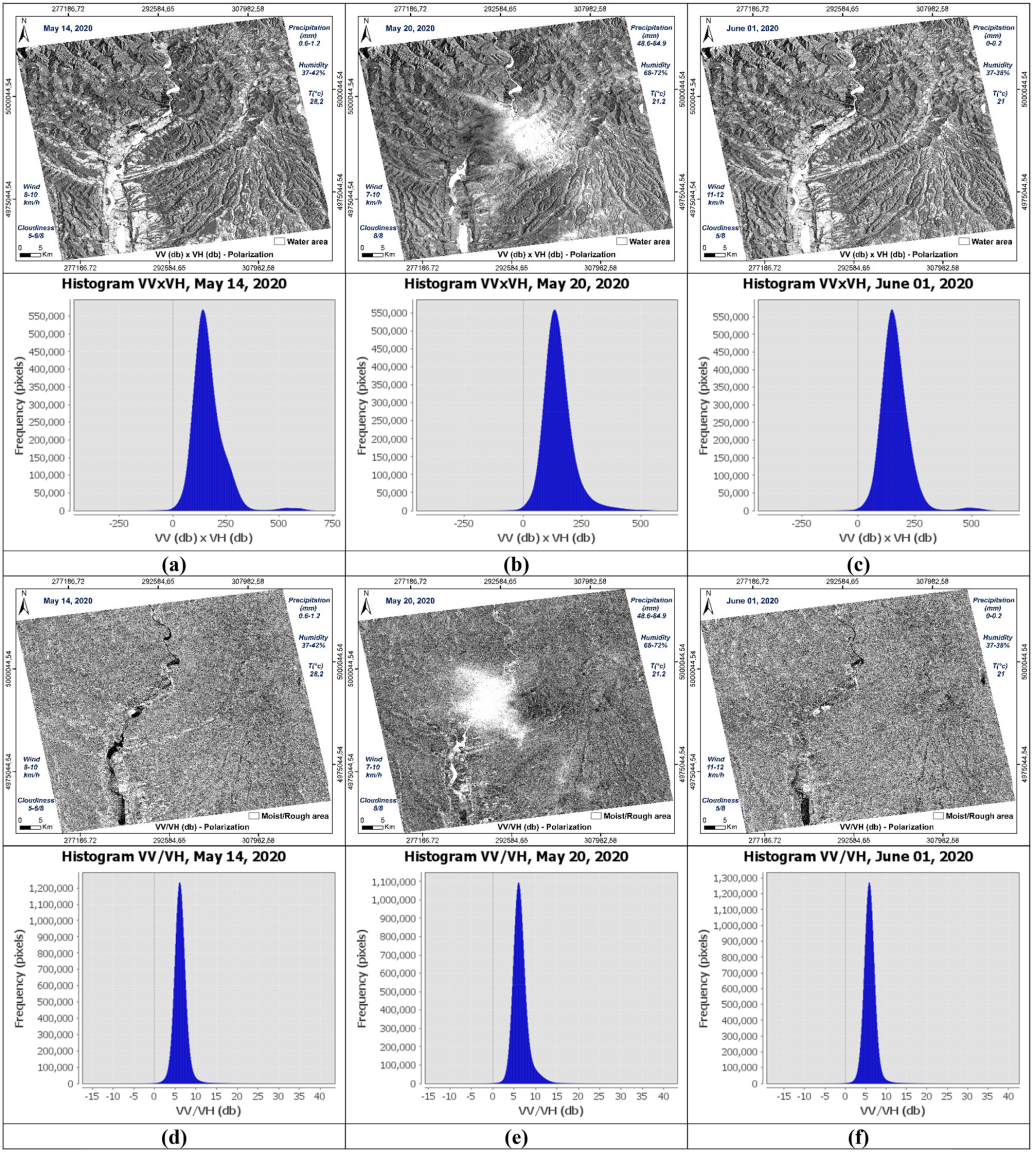
Histograms of the multiplication and division of the two polarizations for each date of the three SAR images used, before (14/05/2020), during (20/05/2020) and after (01/06/2020) the downpour. Product VV × VH (**a**–**c**) and VV/VH ratio (**d**–**f**).

The respective histograms of the VV × VH products and the VV/VH ratios of each date are shown in Fig. 12. The VV × VH product, histogram (b) (Fig. 12) is clearly distinguished from the other two (a) and (c) (Fig. 12) thus marking the impact of the downpour of May 20, 2020. The pixels covered with water are well discriminated in the three images and they are more numerous in the histogram corresponding to image (b) (Fig. 12). This is due to the high rainfall recorded (48.6–84.9 mm). On the other hand, the water pixels in the histogram (a) (Fig. 12) are a little more numerous than in the histogram (c) (Fig. 12) because May 14, 2020 recorded a quantity of approximately 0, 6–1.2 mm against 0.2 mm on June 01, 2020. The same observations concerning the VV/VH ratios where wet and rough pixels are more important and well discriminated on May 20, 2020 (Fig. 12e) unlike on the other two dates. Between the two dates, before and after the downpour, there is not a big difference because of the metrological conditions recorded which show a little more moisture in the date before (May 14, 2020). The two dates respectively recorded temperatures of 28.2 °C, precipitation between 0.6 and 1.2 mm and wind speed between 8–10 km/h on May 14, 2020 against temperatures of 21 °C, precipitation between 0 and 0.2 mm and wind speed between 10 and 11 km/h on June 01, 2020 (Tables 4, 5), which shows that wet and rough pixels are a few more in the date of May 14, 2020 than the date of June 01, 2020 because of this small difference in the amount of precipitation (Fig. 12d,f). Table 8 shows the values ​​of **σ0**_**VV**_, **σ0**_**VH**_ and their operations calculated from the control points, 1, 2, 3 and 4, used in Fig. 11.

**Table 8:** Water and moisture/roughness extraction (**WE**_**D**_ and **MRE**_**D**_) as well as change detection (**WCDI**_**D**_ and **MRCDI**_**D**_) of the four points of the representative locations of the scene.

	Geographical coordinates	Acquisition dates									
		14/05/2020			20/05/2020			01/06/2020			20/05/2020
		Backscatter (σ0) Intensity (db)		WE_D_	Backscatter (σ0) Intensity (db)		WE_D_	Backscatter (σ0) Intensity (db)		WE_D_	Downpour
		Polarization			Polarization			Polarization			
Points	Lon/Lat	σ0_VV_	σ0_VH_	σ0_VV_ × σ0_VH_	σ0_VV_	σ0_VH_	σ0_VV_ × σ0_VH_	σ0_VV_	σ0_VH_	σ0_VV_ × σ0_VH_	WCDI_D_
Point 1 ()	24.32°/45.03°	− 22.015	− 26.637	586.41462	− 4.946	− 18.779	92.8.844542	− 18.499	− 24.193	447.53876	0.08983
Point 2 ()	24.39°/45.14°	− 20.438	− 24.807	507.01129	− 18.429	− 23.074	425.230926	− 21.904	− 24.851	544.33102	0.40446
Point 3 ()	24.25°/44.93°	− 23.795	− 26.780	637.23209	− 17.366	− 24.208	420.405848	− 19.450	− 24.175	470.22032	0.37962
Point 4 ()	24.44°/45.03°	− 8.530	− 15.356	130.99427	− 20.177	− 23.372	471.587827	− 8.088	− 13.760	111.29469	1.94639

Points	Lon/Lat	Sigma (σ0)		MRE_D_	Sigma (σ0)		MRE_D_	Sigma (σ0)		MRE_D_	Downpour
		Polarization			Polarization			Polarization			
		σ0_VV_	σ0_VH_	σ0_VV_/σ0_VH_	σ0_VV_	σ0_VH_	σ0_VV_/σ0_VH_	σ0_VV_	σ0_VH_	σ0_VV_/σ0_VH_	MRCDI_D_
Point 1 ()	24.32°/45.03°	0.00734	0.00251	2.92430	0.34825	0.01305	26.68582	0.01515	0.00304	4.98355	3.37459682
Point 2 ()	24.39°/45.14°	0.01210	0.00316	3.82911	0.01854	0.00403	4.600496	0.00653	0.00437	1.49428	0.86420375
Point 3 ()	24.25°/44.93°	0.00402	0.00179	2.24581	0.01579	0.00227	6.955947	0.01360	0.00376	3.61702	1.18644845
Point 4 ()	24.44°/45.03°	0.12011	0.02184	5.49954	0.01109	0.00466	2.379828	0.11498	0.02568	4.47741	0.2385325

**Figure 13:**
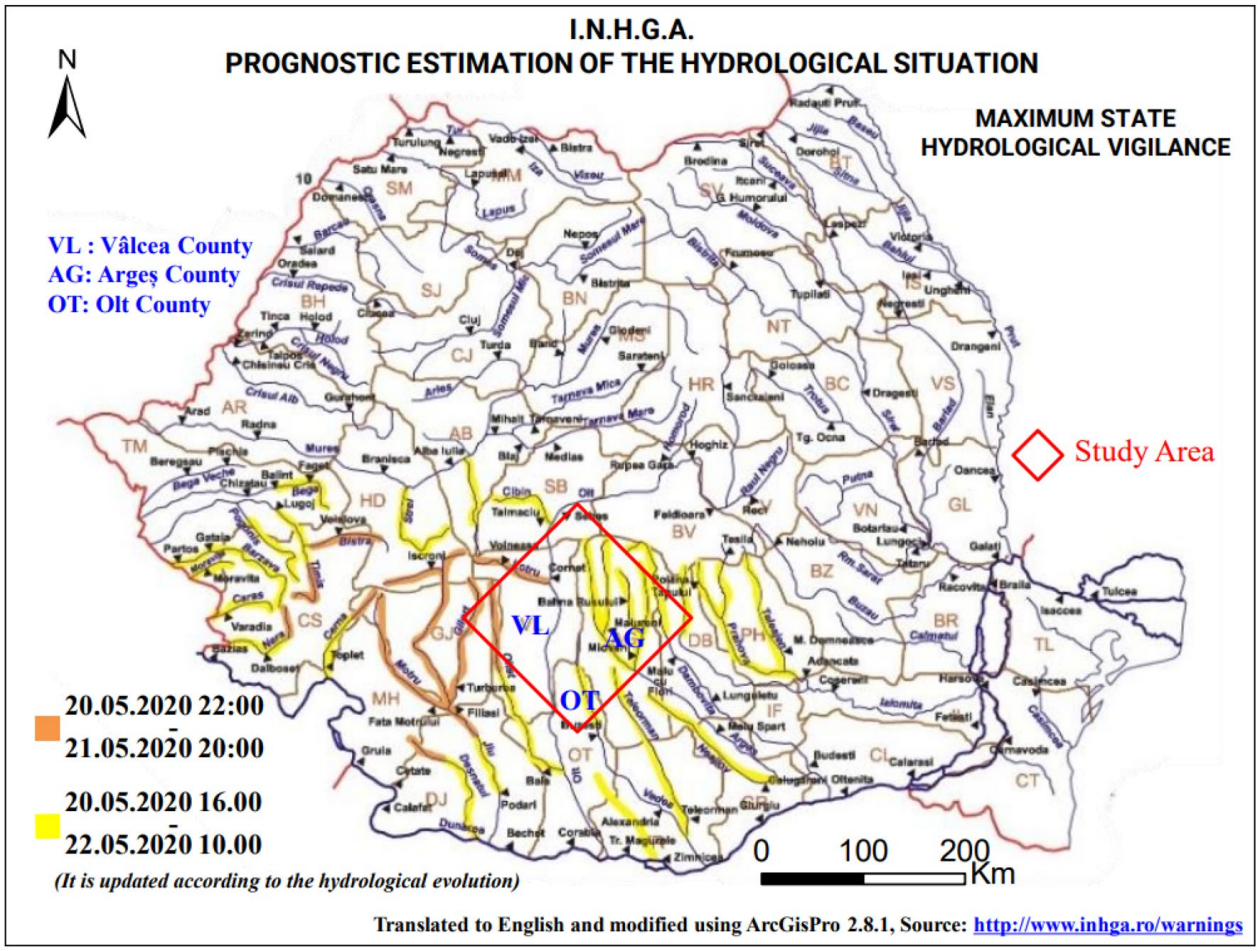
Hydrological warning map, translated to English and modified using ArcGisPro 2.8.1, showing rivers in flood on May 20, 2020: yellow—first warning level and orange—second warning level (out of three). Source: http://www.inhga.ro/warnings.

In this period of May 20, 2020, the area of ​​Vâlcea, marked by a temperature of 21 °C, was covered with stormy clouds, with a high water content which triggered downpours. The weather station of Râmnicu Vâlcea recorded a maximum cloudiness of 8/8, humidity between 68% at 4 p.m. and 72% at 5 p.m. (Table 5) and rainfall of 48.6 mm (Table 6). These downpours are also characterized by hail falling. According to the hydrological alert map which shows the rivers in flood, this area does not record any measured watercourse except the Olt River which did not undergo too great a flood (Fig. 13). This lets us think and interpret the results obtained corresponding to the two markers of the backscattering coefficient, weak and strong, as follows. Areas of weak backscatter signal correspond to areas completely covered with water and areas of high backscatter signal intensities correspond to areas of high roughness due to the interaction of water drops with the soil and also the presence hail, influenced by the wind factor, characterized by a maximum speed of 16 km/h in a northeast direction (Table 5). The influence of the wind factor on the roughness was greater in the open areas which are located in the plains and less (weak) in the areas protected by trees, located on the hills.

This first assumption (hypothesis) is based on the NMA (National Meteorological Administration) weather bulletin, from the morning of May 20, 2020 at 9:00 a.m. to May 21, 2020 at 9:00 a.m. The report mentions that during this 24-h period, the weather was generally unstable and cooled. There were heavy rain (downpour) during the day in most of Oltenia, and in the evening and at night in the south and south-east of the region. The rains were also torrential, and the quantities of water that fell in short time intervals or accumulated exceeded 50–60 l/m^2^ in the hilly area and locally in the Southern Carpathians, up to 86.0 l/m^2^ in Nistoreşti (Gorj County), and in the mountains of 82.1 l/m^2^ at Horezu 1550 m. There were frequent electric discharges and isolated squalls were reported (Turcinești—Gorj County). Small and medium hail were recorded, according to observation data collected from meteorological and hydrological stations, in the counties of Mehedinți, Gorj, Dolj, Vâlcea, Argeș, Dâmbovița, Teleorman, and from external sources and in the counties Olt and Prahova^36^.

Typically, hail forms during the summer months during a thunderstorm. It occurs from the raindrops that form at the bottom of clouds during a thunderstorm. Updrafts during a severe storm carry these raindrops from cloud bottoms to cloud tops, where the temperature is cooler. This cooled water will freeze on contact with ice crystals, dust or other matter and form a tiny piece of hail. The latter then falls to the bottom of the cloud where it will again be carried upwards by an updraft. It will once again be in contact with even colder water which will cause another layer to freeze around the hail, which will also explain the different sizes of the hail. This system will reproduce itself until the updraft weakens or the weight of the hail increases, which is able to heavier in the cloud and eventually fall to the ground.

**Figure 14:**
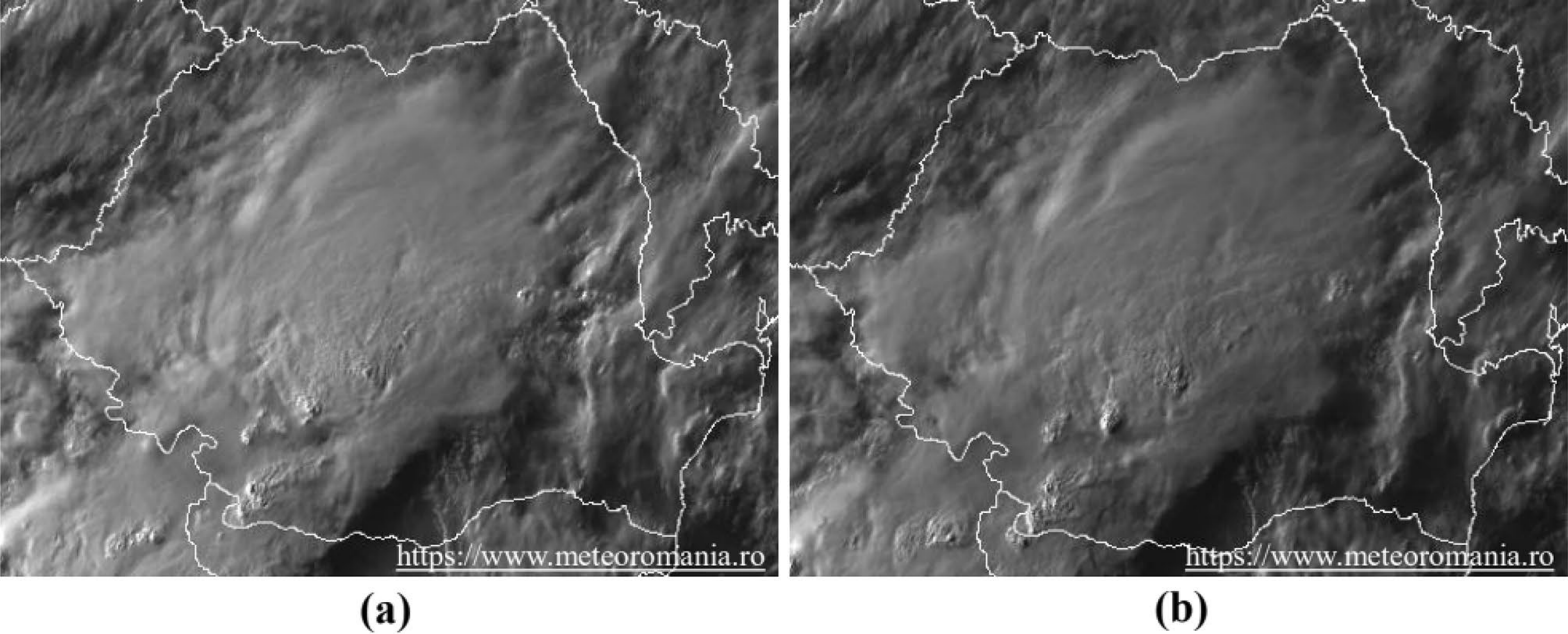
Image showing clouds, acquired on May 20, 2020, (**a**) at 4:15 p.m. and (**b**) at 4:30 p.m. Source: https://www.meteoromania.ro.

**Figure 15:**
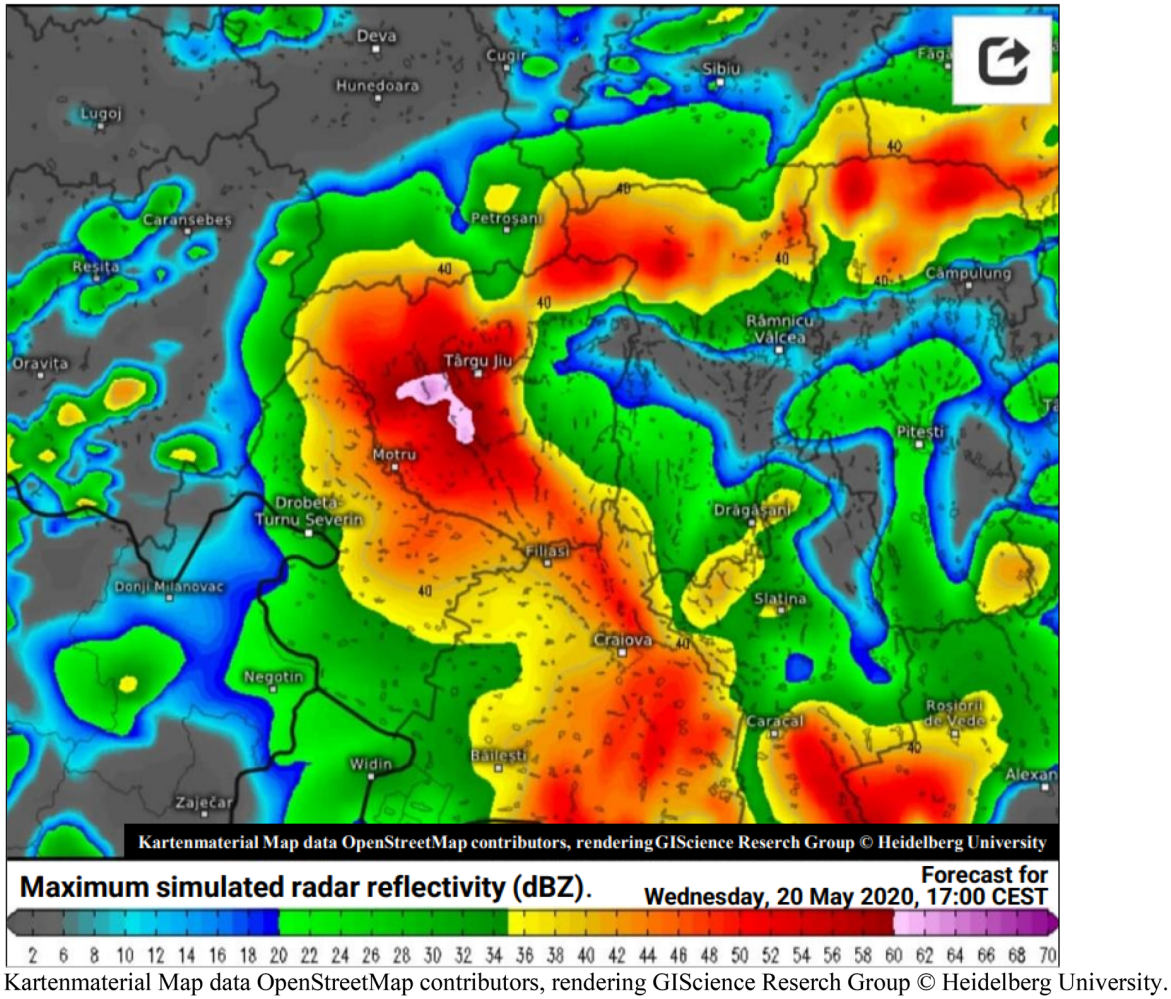
Map translated to English, showing Radar image acquired on 20/05/2020 at 17:00. Colors indicate simulated maximum radar reflectivity (dBz). Raster map 23.5 E, 44.9 N (zoom level 4 / resolution 400 m). Europe Swiss HD 4 X 4 FROM 20 May 2020/00 z. Updates: approximately 7:45 a.m.–11:15 a.m., 1:45 p.m.–5:15 p.m., 7:45 p.m.–11:15 p.m. and 1:45 a.m.–5:15 a.m. Source : https://kachelmannwetter.com/de/info/niederschlagsradar.

**Figure 16:**
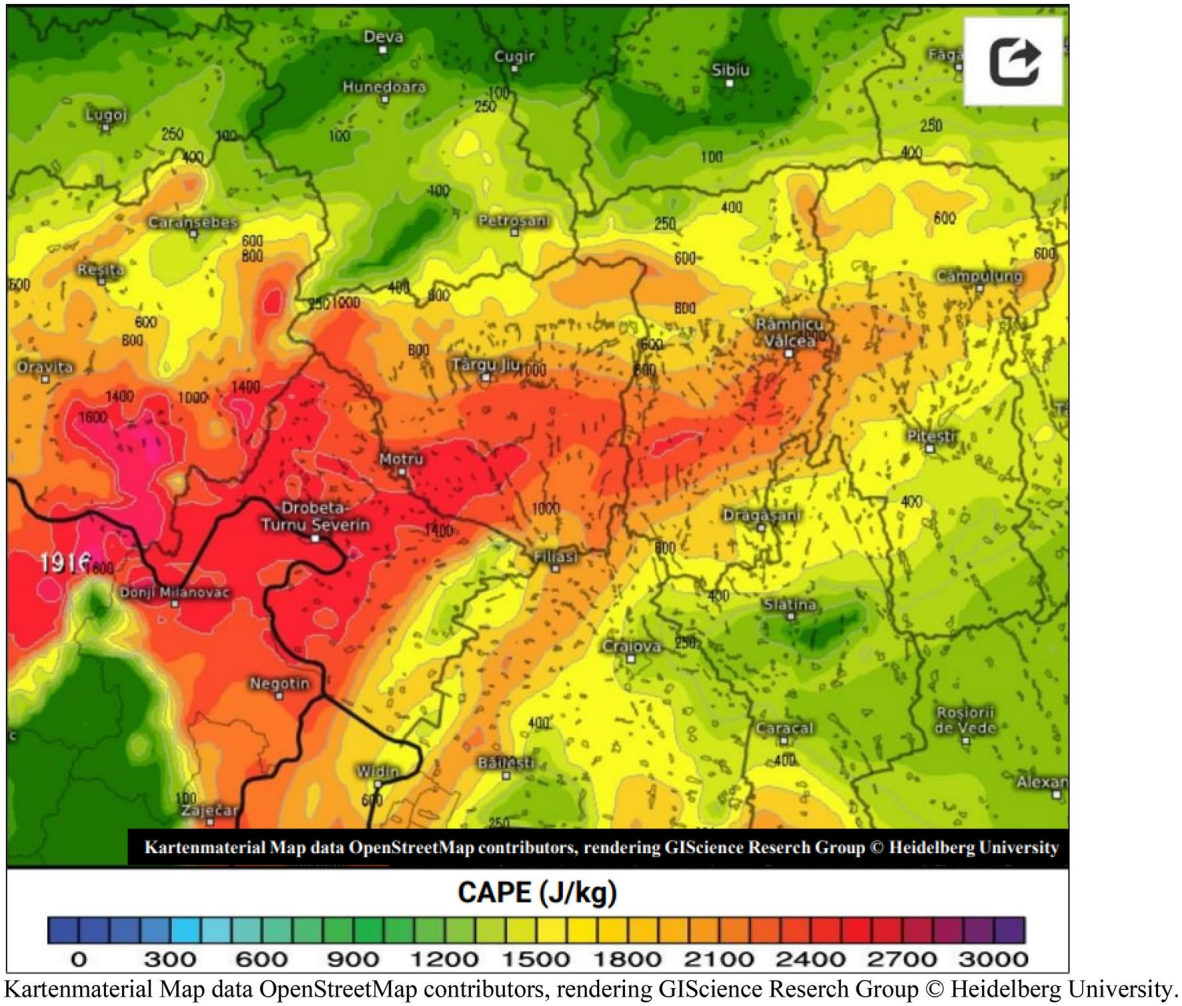
Map showing the CAPE instability index acquired on 20/05/2020 at 17:00. Colors indicate instability index CAPE. Source: https://kachelmannwetter.com/de/info/niederschlagsradar.

The second hypothesis conjectures that the part of strong signal intensity, recorded on May 20, 2020 at 4:17 p.m. by the SAR imaging radar, is due to a thunderstorm cloud, precisely a Cumulonimbus, which formed in this location. Its elongated shape, its calculated area of approximately 36.54 km^2^ (Table 7) and its high water content may suggest that it is at the origin of this high intensity. This very high humidity, characterized by a large dielectric constant, can backscatter the polarization radar wave through VV. Figure 14 shows the clouds over the regions of Romania. On the other hand, Fig. 15 shows the degree of reflectivity (dBZ), recorded in this period of May 20, 2020 and Fig. 16 shows the instability index CAPE (Convective Available Potential Energy) describing the instability of the atmosphere and thus providing an approximation of the strength of the updraft in a thunderstorm. A higher value of this index (CAPE) means that the atmosphere is more unstable and would therefore produce a stronger updraft^37^. Due to the lack of data, it is difficult to confirm with exactitude.

The geomorphology of the study area, the metrological conditions and the anvil shape characterizing the Cumulonimbus, favor this second hypothesis. The place where this high intensity part is located is in the plain, bounded to the northeast by a hill. Following the high humidity and temperature recorded, it is possible that a mass of hot and humid air, rose in the atmosphere by meeting the relief under the effect of the wind direction North-East and maximum of 16 km/h (Table 4).

Cumulonimbuses are characterized by a summit containing ice crystals and vertical extensions can range from 300 m to 17,000 m elevation. They are made up of water droplets and ice particles. They are sources of thunderstorms, extremely heavy downpours, hail, tornadoes and very severe bad weather.

In order to extract and map surfaces entirely or almost covered with water, several methods and approaches have been proposed by exploiting the properties of the SAR radar wave of the recorded signal. Many studies apply thresholding from the numerical values ​​of the backscatter coefficient for a single or each separate polarization, then come the steps using the comparison of the accuracies of the polarizations to delimit and refine the different results, such as^7,9,12–22^. Other works have used indices by calculating them with only one polarization, i.e. VV or HH or VH or HV, without combining them, then afterwards they use thresholding followed by comparisons of these polarizations in order to have precision on the obtained results, such as the following indices: the NDSI (Normalized Difference Scattering Index) adapted to water^38,39^; the NDR (Normalized Difference Ratio) or NDCD (Normalized Difference Change Detection)^40–42^; the NDFI (Normalized Difference Flood Index) and the NDFVI (Normalized Difference Flood short Vegetation Index)^43–45^; the NCI (Normalized Change Index) and the RI (Ratio Image)^10,11^; the NoBADI (Normalized Backscatter Amplitude Difference Index)^46^; the UFI (Urban Flooding Index)^47^; the RI (Ratio Image)^48^.

On the other hand, the method used in this work differs by using and combining in the calculation operations the two backscattering coefficients of the two polarizations VV and VH without comparing them thereafter.

In view of all these different particular situations described, it is very difficult to make a faithful comparison because it is about the impact of a very intense phenomenon that is the Cumulonimbus and the hypothesis of a case study rare where the radar wave has difficulty penetrating a cloud. This can cause problems in interpreting recorded SAR radar images in these extreme cases. However, the approach chosen in this study already includes and encompasses the different polarizations in the calculations, which means that the comparisons between these two quantities are already integrated. This advantage makes it possible to eliminate or minimize any errors that may be accumulated by multiplying the steps. This greatly facilitates analysis and interpretation.

**Figure 17:**
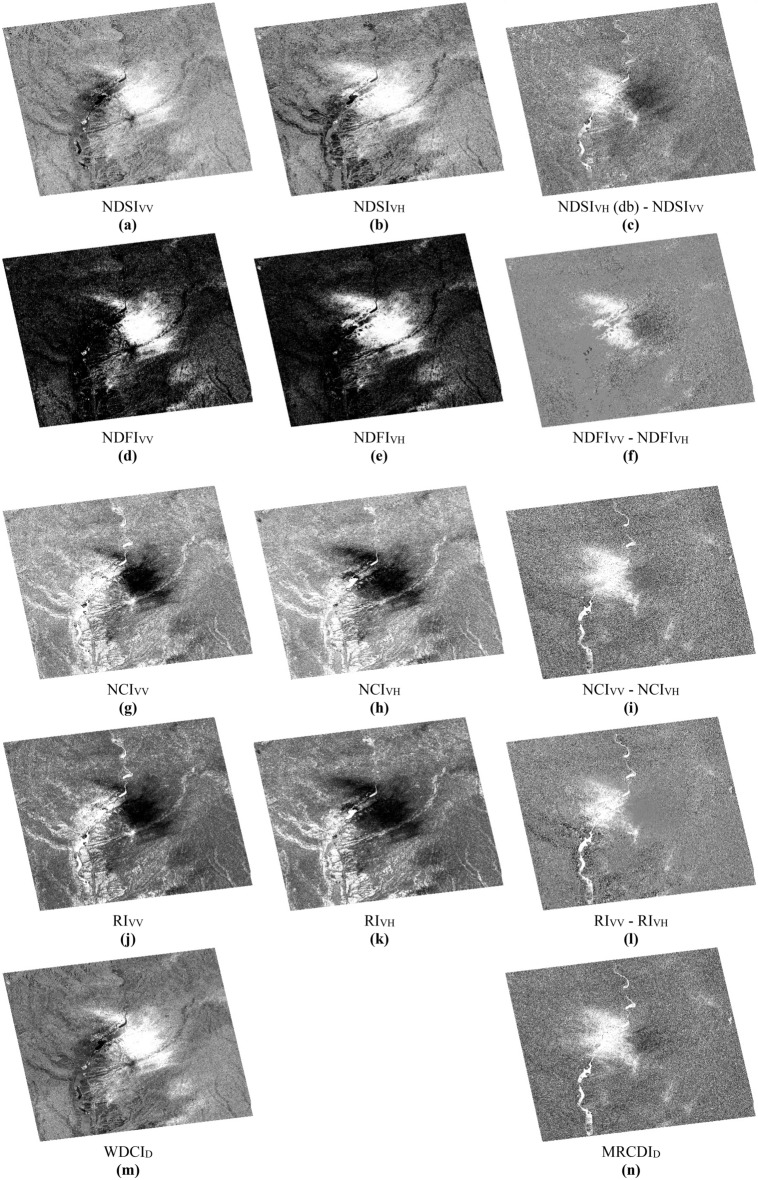
Images showing the results of all the indices tested as well as those used in this study; (**a**–**c**) NDSI index with its two components and their subtraction; (**d**–**f**) NDFI index with its two components and their subtraction; (**g**–**i**) NCI index with its two components and their subtraction; (**j**–**l**) index RI with its components and their subtraction; (**m**) WDCI_D_ index; (**n**) MRCDI_D_ index.

The method proposed in this work was compared to other different indices that have been used in the detection of water-covered surfaces following floods such as the NDSI (Normalized Difference Scattering Index)^38,39^; the NDR (Normalized Difference Ratio)^40–42^; the NDFI (Normalized Difference Flood Index)^43–45^; the NCI (Normalized Change Index) and the RI (Ratio Image)^10,11,48^; the NoBADI (Normalized Backscatter Amplitude Difference Index)^46^. The first constraint encountered, concerns the choice of the band used, because all these indices use a single band, either VV or VH and the second will be used and will help to define a threshold but they prefer the VV band which is more sensitive to soil moisture and roughness than the VH band. This requires us to use the two polarizations VV and VH for each index tested on our images in order to have a good analysis of the results (Fig. 17). Compared to the proposed index **WDCI**_**D**_ (Fig. 17 (m)), this comparison shows almost the same results in the detection of surfaces covered with water during the downpour of May 20, 2020 (Fig. 17a,d,g,j). Regarding the wet and rough areas, detected using the **MRCDI**_**D**_ index (Fig. 17n), we performed subtractions between the two results of each tested index of each band used (Fig. 17c,f,i,l). This also means that the results of these indices contain wet or rough pixels to be taken into consideration in order to keep only the water pixels. We also tested the NDR index^40,41^ but the results are not presented because it uses the same principle of normalization as the NDSI index. That is to say, it uses the ratio of the subtraction between two images of amplitudes (σ0) acquired before and after the incident on the addition of these same two images (σ0) for a single band, either VV or VH. Regarding the NoBADI index^46^, no result was obtained. Furthermore, the UFI index^47^ was not tested in this study because its combination uses coherence images derived from InSAR interferometry, i.e., a band of temporal source which concerns the phase of the signal.

The differences between these various methods and ours can be summed up by the fact that the indices tested use a single reference date and a single band. On the other hand, our proposed method uses two reference dates and it exploits by combining the two bands of the backscattered signal VV and VH, therefore more support and information. It can also use several reference images by dividing by their sum ($\sum_{{\varvec{i}}}^{{\varvec{n}}}{{\varvec{\upsigma}}0}_{{\varvec{V}}{\varvec{V}}\times {\varvec{V}}{\varvec{H}}}{(i)}$ for Water and $\sum_{{\varvec{i}}}^{{\varvec{n}}}{{\varvec{\upsigma}}0}_{{\varvec{V}}{\varvec{V}}/{\varvec{V}}{\varvec{H}}}{(i)}$ for Moisture/Roughness) for Moisture/Roughness) where i:  corresponds to the dates of the reference images used and n: corresponds to the numbers of the reference images used.

## Conclusion

This study allowed us to discriminate and extract the impact of the downpours and their origin Cumulonimbus produced on May 20, 2020, using combinations of the two polarizations, VV and VH, of the radar wave of the Sentinel-1 series. It allowed us to distinguish between surfaces completely covered with water and rough surfaces due to falling hail and the interaction of water drops with the ground, increased by the wind factor or a moist surface due to its water content located in the cumulonimbus. The latter is characterized by a strong backscattering signal, discriminated by the parallel polarization, VV. On the other hand, the first surface is represented by a very low backscattering coefficient signal, distinguished by the cross polarization, VH. It also allowed us to estimate the areas of these two different areas affected by these downpours. This study also showed the importance and advantage of cross-polarization, VH in determining water-covered surfaces during weather events and also its help in discriminating rough and wet surfaces by combining it with parallel polarization, VV.

This work also represents a very rare case where the 'C' band SAR wave fails to penetrate clouds. It demonstrates that high density Cumulonimbus clouds can be one of the limitations of SAR imagery but it can also detect and map them.

## Data Availability

All data produced from this study are provided in this manuscript.
